# Binding Site Vectors Enable Mapping of Cytochrome P450 Functional Landscapes

**DOI:** 10.1021/acs.jcim.5c02705

**Published:** 2026-01-26

**Authors:** Tea Kuvek, Zuzana Jandová, Klaus-Juergen Schleifer, Chris Oostenbrink

**Affiliations:** † Institute for Molecular Modeling and Simulation, BOKU University, Muthgasse 18, 1190 Vienna, Austria; ‡ Christian Doppler Laboratory for Molecular Informatics in the Biosciences, BOKU University, Muthgasse 18, Vienna 1190, Austria; § Boehringer Ingelheim International GmbH, Dr.-Boehringer-Gasse 5-11, 1121 Vienna, Austria; ∥ BASF SE, Carl-Bosch-Strasse 38, 67056 Ludwigshafen, Germany

## Abstract

Understanding similarities between protein binding sites has long been of great interest, as such comparisons can reveal functional relationships that transcend sequence or fold. However, systematic comparison remains challenging due to the difficulty of defining active sites consistently and developing descriptors that are both general and discriminative. We present *binding site vectors*, a computational framework for a high-resolution comparison of macromolecular binding sites that integrates both structural and electrostatic properties. The vectors extend spherically from the center of the pocket, terminating at its surface to capture shape and electrostatic features in a multidimensional manner. Geometrically anchored, they enable a systematic comparison of binding sites across diverse systems. We applied this approach to cytochrome P450 (CYP) enzymes, analyzing over 600 human and plant CYP structures and a subset of 23 extensive structural ensembles obtained through molecular dynamics (MD) simulation. Comparisons based on binding site vectors reveal structural–functional relationships missed by sequence- or backbone-based groupings, particularly when full conformational ensembles are included. This demonstrates that binding site vectors provide a robust framework for both functional classification and deep mechanistic insights into macromolecular systems.

## Graphical abstract

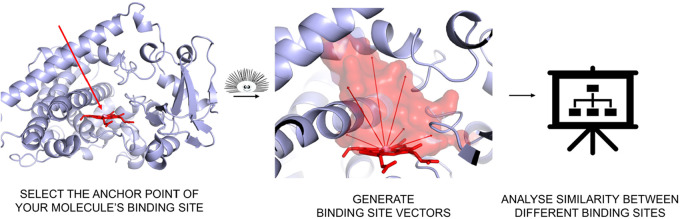

## 1 Introduction

The shape and physicochemical properties of the binding pockets are key determinants of molecular recognition between macromolecules and their ligands. This principle is particularly pronounced in systems where ligand binding follows the mechanism of conformational selection, where conformational protein changes happen prior to binding. In such cases, the structural flexibility of the binding site can enable recognition of a broader range of ligands, whereas more rigid macromolecules tend to display higher binding specificity.^1,2^

Predicting such binding behavior can be achieved by systematic characterization of binding sites, which has therefore long been a standard approach, especially for enzymes and pharmacologically relevant targets. Consequently, numerous computational tools were developed for this purpose and are collected in a review by Utgés and Barton.^3^ These tools provide functionalities, such as identification of potential pockets, estimation of their druggability scores, and quantification of key properties such as volume, surface area, and polarity. While existing methods provide valuable general descriptions of binding pockets, they offer limited ability to perform high-resolution comparisons that capture subtle differences in topology and physicochemical properties. It remains particularly challenging to systematically compare binding sites of proteins that show a similar overall structure but differ in the exact amino acids aligning the active site. This quickly restricts protein structure-based methods to the conformations of the backbone only, even though the side chains interact most strongly with any putative ligands.

In our previous work, we used the *fpocket* ^4^ and *mdpocket* ^5^ tools, both discussed in the review of Utgés and Barton,^3^ as a part of a computational pipeline for the dynamic characterization of binding pockets.^6^ These analyses provided key descriptors, such as pocket volume, surface area, overall polarity, and shape estimates. Although informative, the results did not enable direct grouping of the studied systems or a detailed assessment of topological similarities between their binding sites. An intuitive extension would be to align proteins based on their backbone and calculate the structural deviation of only active site residues. However, this is not straightforward in practice as it can be difficult to systematically define which residues make up the active site, especially in highly flexible proteins. The challenge grows with sequence divergence in the observed proteins.

In this work, we present *binding site vectors*, a method to address these limitations. Our approach encodes both the surface topology and local electrostatics of a pocket in a multidimensional format. Specifically, a user-defined number of vectors radiate spherically from a chosen binding site center within the macromolecule until they intersect an atomic surface. For each vector, both the final length and partial charge of the encountered atom are recorded. This representation enables systematic, per-vector comparison of binding pockets, uncovering structural and electrostatic features that govern similarities in binding behavior and specificity across studied systems.

To evaluate the performance of the binding site vectors, we applied our method to cytochrome P450 enzymes (CYPs). CYPs constitute a highly diverse superfamily of enzymes found in all kingdoms of life, where they play central role in both endogenous and exogenous metabolism.^7–13^ Their substrate binding behavior is largely governed by conformational selection, as dynamic loops around the heme cofactor enable the accommodation of diverse ligands.^14,15^ Although CYPs are typically classified by sequence similarity, such groupings often fail to capture functional relationships. Furthermore, with the exception of human CYPs, substrate preferences across the superfamily remain poorly defined, underscoring the need for alternative descriptors to guide functional classification.^9,12^ These features make CYPs a compelling test case in which binding site vectors may deliver useful insights into functional organization, capturing both similarities across CYP groups and the extent of variations observed between alternative conformations of the same isoform.

## 2 Methods

In the following section, we introduce the way the binding site vectors are constructed and compared, as outlined schematically in Figure 1. This is followed by a detailed description of their application to the CYP superfamily. We then describe the data sets and the molecular simulations used to generate broad ensembles of a subset of CYPs. We end the methods section with a description of three case studies to demonstrate different uses of the binding site vectors.

**Figure 1:**
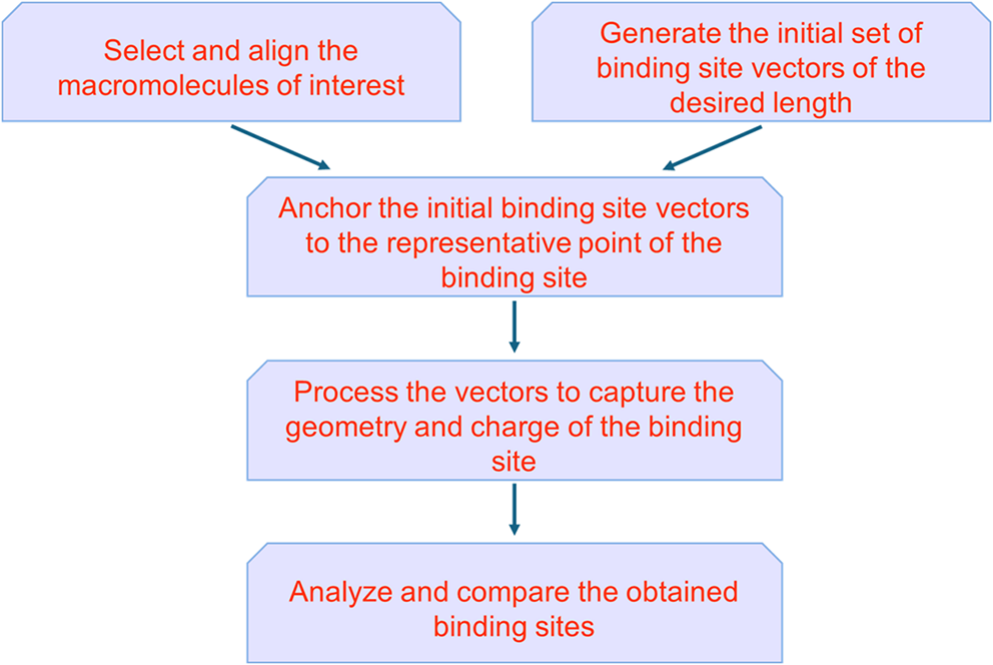
Schematic overview of the binding site vectors methodology.

### 2.1 Binding Site Vectors Generation

The workflow begins, as shown in Figure 2, with a sphere of defined radius, inside which an icosahedron is placed. The icosahedron faces are uniformly subdivided into a defined number of triangles, and the resulting vertices are projected onto the sphere surface, forming a triangular lattice sphere.^16^ The vectors are extended from the sphere center toward the projected vertices, after which the sphere is positioned at the defined center of the binding site under investigation.

**Figure 2:**
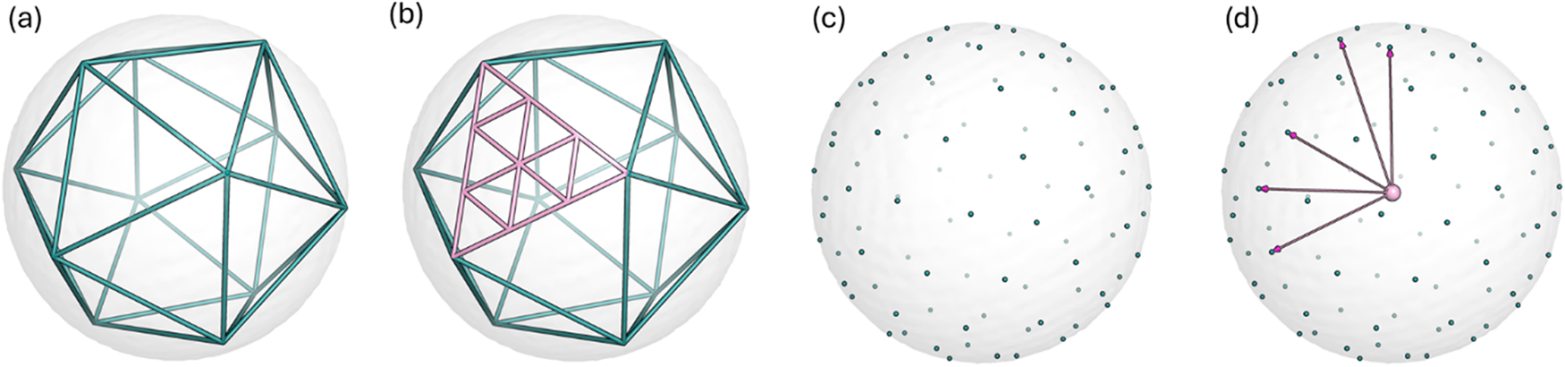
Initial binding site vectors formation: (a) Icosahedron placed inside a sphere, (b) Icosahedron’s triangle faces split into smaller triangles, (c) the vertices of the subdivided triangles projected onto the sphere surface, (d) vectors originating from sphere center toward the vertices.

To capture the geometry and electrostatics of the binding pocket, each vector is traced until it intersects with the surface of the atom that is closest to its origin (Figure 3).

**Figure 3:**
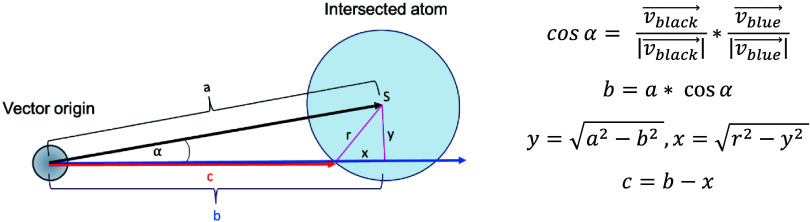
Geometric procedure for determining the intersection between a binding site vector and protein atoms. The black vector points from the binding site origin to the atom center, the blue vector represents the initial vector direction, and the red vector is the truncated binding site vector ending at the atom surface. The corresponding distances are denoted as *a* (black vector), *b* (black vector’s projection on initial vector), and *c* (red vector), which are used in the calculations.

For every atom in the protein, the perpendicular distance between the atom center and the binding site vector is calculated (corresponding to the *y* value in Figure 3). If this distance exceeds the atom’s van der Waals radius, the binding site vector does not intersect the atom; if the distance is equal to or smaller than the radius, an intersection occurs. Using the equations shown in Figure 3, the new vector length (*c*) is then determined, reaching the point of intersection. For all atoms intersected by a given binding site vector, *c* is calculated, and the atom with the smallest *c* value is identified as the intersection point. Finally, the binding site vector length is set to this *c* value, and its charge is assigned as the partial charge of the intersected atom. If no atom is encountered, the vector retains the initial sphere’s radius as its length, and its charge is set to zero.

### 2.2 Comparison of Binding Sites

The similarity between two binding sites can be evaluated based on shape, electrostatics, or both. In all cases, the length and/or charge of each vector is compared, and the average of the resulting differences is calculated. Comparisons are performed exclusively between the corresponding vector indices. To ensure meaningful results, the macromolecules under study must be aligned so that each vector of a given index points toward the intended binding site region.

The root-mean-square difference (RMSD) between two binding sites *i* and *j* based solely on shape is calculated as

1 $${\mathrm{RMSD}}_{\mathrm{ij}}^{s}=\sqrt{\frac{1}{N}\sum_{k=1}^{N}{\left(l_{k,i}-l_{k,j}\right)}^{2}}$$

where superscript *s* stands for shape, *N* is the total number of vectors, and *l*_*k,i*_ and *l*_*k,j*_ are the lengths of vector *k* for binding sites *i* and *j*, respectively.

The difference based solely on charge is calculated as

2 $${\mathrm{RMSD}}_{\mathrm{ij}}^{c}=\sqrt{\frac{1}{N}\sum_{k=1}^{N}{\left(q_{k,i}-q_{k,j}\right)}^{2}}$$

where superscript *c* stands for charge and *q*_*k,i*_ and *q*_*k,j*_ are the partial charges of vector *k* for binding sites *i* and *j*, respectively.

To assess differences based on both shape and electrostatics, vector lengths and partial charges are normalized to make them comparable and unitless:

3 $${RMSD}_{\mathrm{ij}}^{\mathrm{sc}}=\sqrt{\frac{1}{N}\left[\sum_{k=1}^{N}{\left(\frac{l_{k,i}-\mathrm{l̅}}{\sigma_{l}}-\frac{l_{k,j}-\mathrm{l̅}}{\sigma_{l}}\right)}^{2}+\sum_{k=1}^{N}{\left(\frac{q_{k,i}-\mathrm{q̅}}{\sigma_{q}}-\frac{q_{k,j}-\mathrm{q̅}}{\sigma_{q}}\right)}^{2}\right]}$$

where *l̅* and σ_l_ are the mean and standard deviation of all vector lengths, and *q* and σ_q_ are the mean and standard deviation of all partial charges. Simplifying eq 3 gives

4 $${\mathrm{RMS}D}_{\mathrm{ij}}^{\mathrm{sc}}=\sqrt{\frac{1}{N}[\sum_{k=1}^{N}{\left(\frac{l_{k,i}-l_{k,j}}{\sigma_{l}}\right)}^{2}+\sum_{k=1}^{N}{\left(\frac{q_{k,i}-q_{k,j}}{\sigma_{q}}\right)}^{2}]}$$

Furthermore, weights can be introduced to tune the relative contributions of shape and charge to the overall RMSD:

RMSDijsc(ws,wc)=1N[ws∑k=1N(lk,i−lk,jσl)2+wc∑k=1N(qk,i−qk,jσq)2],ws+wc2=1 5

Here, the weights *w*_*s*_ and *w*_*c*_ correspond to the contributions of shape and charge, respectively, and are related through a tuning parameter *d* as

6 $$w_{c}=\left(1+d\right)*w_{s},w_{s}=\frac{2}{2+d},\;w_{c}=\frac{2\left(1+d\right)}{2+d}$$

By adjusting the parameter *d*, one can place greater emphasis either on the shape component (*d* < 0) or the electrostatic component (*d* > 0), providing flexibility to prioritize the molecular features most relevant to a given analysis.

### 2.3 Binding Site Vectors for Cytochrome P450s

In this study, the binding site vector methodology was applied to cytochrome P450 enzymes with the binding site located above the planar heme group (Figure 4a). The iron atom at the center of the heme served as an anchor point for the binding site vectors. From this anchor, 492 vectors with a maximal length of 20 Å were generated (Figure 4b). To focus on the relevant binding region, the vectors were restricted to the hemisphere extending above the heme, reducing the set to 260 vectors (Figure 4c). Following the procedure outlined in Section 2, the processed vectors represent the final binding site, as exemplified in Figure 4d. When processing the length of the vectors, the heme group was not considered to avoid extremely short vectors.

### 2.4 Cytochrome P450 Data Sets

The full list of observed human and plant CYPs with their corresponding PDB IDs and UniProt codes can be found in Tables S1 and S2, respectively.

#### 2.4.1 Human CYPs for Single Structure Observation

A set of human cytochrome P450 structures was obtained by retrieving all PDB entries with the full name “Cytochrome P450” that met the following criteria: (i) *Homo sapiens* as the source organism, (ii) single protein entity, (iii) presence of a heme group with the iron atom positioned within 4 Å of any cysteine sulfur, and (iv) full C_α_ RMSD ≤ 7 Å relative to the reference CYP3A4 structure (PDB ID: 4I3Q^17^). The final RMSD filter was chosen empirically, as the initially filtered set still included some non-CYP structures. Missing residues were reconstructed using PDBFixer,^18^ monomers were extracted from multimeric assemblies, and any existing ligands and waters were removed. Applying these criteria resulted in 285 human CYP structures, representing 25 distinct CYPs across 11 families, with resolutions ranging from 1.4 to 3.9 Å.

#### 2.4.2 Plant CYPs for Single Structure Observation

For plant CYPs, all AlphaFold2-predicted structures with protein names containing “Cytochrome P450” were retrieved from the UniProt database^19^ and filtered according to the following criteria: (i) *Viridiplantae* as the source organism, (ii) structures verified by SwissProt, and (iii) full C_α_ RMSD ≤ 7 Å relative to the reference CYP3A4 structure (PDB ID: 4I3Q). This selection yielded 343 plant CYP structures from 45 distinct families, predicted with AlphaFold2.^20^ Given the extensive structural data available for CYPs and the conserved nature of their fold, the collected AlphaFold2 models exhibit pLDDT confidence scores ranging from high to very high confidence, supporting the reliability of these predicted structures for subsequent analysis.

#### 2.4.3 CYPs Studied with MD Simulations

This data set comprised 23 CYP isoforms. Of these, 18 CYPs (15 plant and 3 human) had been simulated in our previous study,^6^ with the resulting trajectories utilized here. The plant subset includes CYPs from rice (*Oryza sativa*), corn (*Zea mays*), potato (*Solanum tuberosum*), sorghum (Sorghum bicolor), sea arrowgrass (*Triglochin maritima*), and thale cress (*Arabidopsis thaliana*). MD simulations were performed for five additional human CYPs, starting from the experimentally obtained structures listed in the Protein Data Bank: CYP2C9 (PDB ID: 5W0C^21^), CYP2C19 (PDB ID: 4GQS^22^), CYP2D6 (PDB ID: 2F9Q^23^), CYP17A1 (PDB ID: 3RUK^24^), and CYP19A1 (PDB ID: 3S79 ^25^). Missing residues were reconstructed using PDBFixer, and any ligands present in the structures were removed prior to the simulation.

### 2.5 MD Simulation Setup

System preparation and simulation settings were adopted from our prior study.^6^ Briefly, MD simulations were performed with GROMOS^26^ and Gromacs[Bibr ref27] (2020 version) simulation engines using the GROMOS 54a8 force field^28^ and simple point charge (SPC) water.^29^ Each system was simulated in five independent replicas for 500 ns, yielding a cumulative simulation time of 2.5 μs. A total of 15,625 conformations per isoform were extracted to assess conformational differences at both whole-protein and binding site levels, with simulation waters and ions removed prior to analysis.

### 2.6 Structural Alignment and Vectors Generation

For a meaningful comparison, all structures retrieved from databases and conformations generated from MD simulations were aligned using backbone atoms prior to binding site analysis. One reference structure (CYP3A4, PDB ID: 4I3Q) was first translated and rotated so that the heme lays in the xy-plane with the iron atom at the coordinate origin. Database CYP structures were aligned to this reference. For plant CYPs, which lack heme in their AlphaFold2-predicted models, the iron atom was assumed to be at the coordinate origin following alignment. For simulated CYPs, the first frame of each isoform’s trajectory was aligned to the reference and translated to position their heme iron at the coordinate origin as well. The subsequent frames were then aligned to the first one using residues identified as stable secondary structural elements by the *GROMOS++ program*;^30^ only residues present in secondary structure in more than 97% of the simulation were used for alignment. All alignments described were performed using the *PyMol* ^31^ align algorithm. Binding site vectors for all structures were anchored at the coordinate origin, and their lengths and charges were calculated as described in Section 2.1 For each structure, the atoms at the vector end points were assigned van der Waals radii via *OpenBabel*,^32^ and the partial charges were assigned based on the GROMOS 54a8 force field.

### 2.7 Clustering of MD Simulation Conformations

#### 2.7.1 Clustering Based on Binding Site Vectors Similarity

Conformations from each simulation were represented by 260 vectors with assigned lengths and charges. Pairwise RMSD matrices for these conformations were constructed based on: (i) shape only (eq 1), (ii) charge only (eq 2), (iii) both properties (eq 4), (iv) both properties with 50% higher contribution to shape (eq 5), and (v) both properties with 50% higher contribution to charge (eq 5). The matrices served as input for clustering with the Affinity Propagation (AP) algorithm^33^ in scikit-learn.^34^ For each CYP isoform, the method produced a set of exemplars (cluster representatives) along with the number of frames assigned to them (weights).

**Figure 4:**
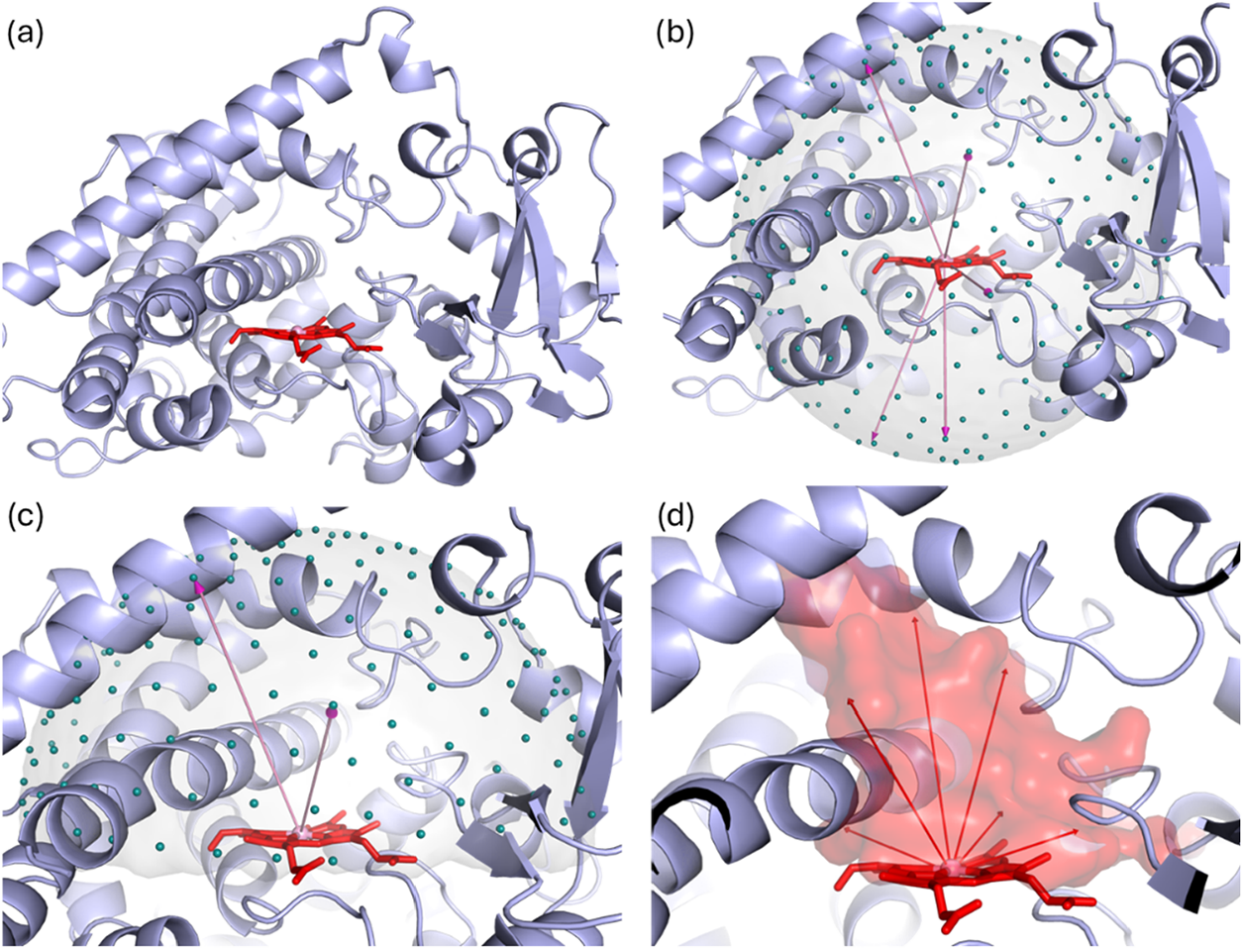
Binding site vectors generation for Cytochrome P450s on the example of CYP3A4 (PDB ID: 4I3Q): (a) structure of CYP3A4 with the heme group shown in red and the central iron atom in pink; (b) initial set of binding site vectors, extending from the heme iron and covering the full sphere; (c) hemisphere of vectors oriented above the heme plane; (d) final binding site shape defined by the processed vectors.

#### 2.7.2 Clustering Based on Full-Structure Similarity

A similar clustering procedure was applied directly to the MD trajectories using MDAnalysis^35,36^ and its Affinity Propagation implementation. Clusters were identified for each isoform based on backbone atom-positional RMSD matrices. The centroid frame of each cluster was extracted as the exemplar, and the number of frames assigned to each cluster was recorded.

### 2.8 Applications/Case Studies

#### 2.8.1 Case Study 1: Structural Similarity of Human (PDB) and Plant (UniProt) Cytochrome P450s Based on Static Structures

The first case study included the structures described in Sections 2.4.1 and 2.4.2, which were aligned according to the protocol in Section 2.6.

For both human and plant CYPs, three types of similarity trees were generated: a phylogenetic tree, a full-structure similarity tree, and a binding site similarity tree. All trees were constructed based on RMSD matrices, with hierarchical clustering average linkage method of Python SciPy package.^37^

The similarity matrices were derived as follows: (i) the phylogenetic tree was based on sequence overlap, with a sequence similarity matrix obtained from multiple sequence alignment of Clustal Omega;^38^ (ii) the full-structure similarity tree was based on a pairwise RMSD matrix obtained from PyMOL’s *cealign* function on all backbone atoms; and (iii) the binding site similarity tree was constructed from a matrix of pairwise RMSD values computed according to eq 4, with σ_l_ = 3.742 Å and σ_q_= 0.198 *e* for human CYPs and σ_l_ = 3.833 Å and σ_q_= 0.213 *e* for plant CYPs. The standard deviations were computed separately from all vector lengths and charges of the observed human and plant CYP structures as the two groups were analyzed independently.

#### 2.8.2 Case Study 2: Ensemble-Averaged Cytochrome P450 Structural Similarity Derived from MD Simulations

In case study 2, as in case study 1, three types of similarity trees were generated: a phylogenetic tree, a full structure-based tree, and a binding site-based tree. The key distinction from case study 1 is that the full structure and binding site trees were derived from the dynamic conformational ensembles obtained from molecular dynamics (MD) simulations rather than from single static structures. All trees were constructed using hierarchical clustering with the average linkage method from the SciPy package.

The phylogenetic tree was based on a sequence similarity matrix obtained from multiple sequence alignment of Clustal Omega, following the approach used in case study 1.

For the full structure and binding site similarity trees, similarity matrices were computed on the exemplars from the clustering protocols from Section 2.7.2 and Section 2.7.1., respectively. RMSD values for the full protein structure were calculated using the *cealign* command in PyMOL. For the binding site similarity, RMSD values were calculated according to eqs 1, 2, 3, 4, and 5 (with σ_l_ = 4.183 Å and σ_q_= 0.214 *e*), depending on the property under consideration (shape, charge, or combined).

To account for the number of simulation conformations represented by each exemplar structure, weighted RMSD matrix values were computed in both cases according to the equation:

7 $$\mathrm{WEIGHTE}{D\_\mathrm{RMSD}}_{i,j}={\mathrm{RMSD}}_{i,j}*\frac{n_{i}}{N}*\frac{n_{j}}{N}$$

where *n*_*i*_ and *n*_*j*_ are the weights of structures *i* and *j*, and *N* is the total number of conformations per simulation (15625).

The overall RMSD between two CYP isoforms, A and B, was then calculated by summing the weighted RMSDs over all pairs of representative structures of the two isoforms:

8 $${\mathrm{RMSD}}_{A,B}=\sum_{i\in A}\sum_{j\in B}{\mathrm{WEIGHTED}\_\mathrm{RMSD}}_{i,j}$$

where *i* ∈ *A* and *j* ∈ *B* indicate representative structures belonging to enzymes *A* and *B*, respectively.

Weighting ensures that structures representing larger portions of the ensemble contribute proportionally more to the similarity assessment. The resulting RMSD matrices were subsequently used to generate the full structure-based and binding site-based similarity trees.

#### 2.8.3 Case Study 3: Binding Site Conformational Diversity and Overlap across Cytochrome P450s Derived from MD Simulations

In case study 3, the exemplars obtained from the clustering of MD simulation conformations based on binding site similarity (described in Section 2.7.1) were combined into a joint data set. A second round of clustering was then performed on this set with the Affinity Propagation algorithm based on RMSD matrices derived from eq 4. This procedure yielded a handful of clusters, each represented by an exemplar structure. The weights assigned during the first clustering were propagated here, making it possible to determine the number of total simulation frames of each CYP that was placed into each cluster in the second round of clustering.

## 3 Results and Discussion

The binding site vector methodology was applied to a data set of over 600 cytochrome P450 enzymes. Across three case studies, the focus was on comparing the grouping of CYPs based on the amino acid sequence, overall backbone structure, and the geometry and pharmacophoric features of the binding site. Each case study is presented with its individual results, highlighting the insights gained from binding site vectors. A subsequent comparison of the case studies evaluates which method of grouping CYPs most accurately captures to their functional similarity

### 3.1 Case Study 1: Structural Similarity of Human (PDB) and Plant (UniProt) Cytochrome P450s Based on Static Structures

The first case study, involving 285 human and 343 plant CYP structures, was performed as detailed in Section 2.8.1. Phylogenetic trees (Figures S1 and S2), backbone similarity-based dendrograms (Figures 5a and S3), and binding site vector-based dendrograms (Figures 5b and S4) were constructed for human and plant CYPs separately. In this section, we focus on human CYPs, outlining plot interpretation and discussing the findings. The same approach can be applied to plant CYPs.

**Figure 5:**
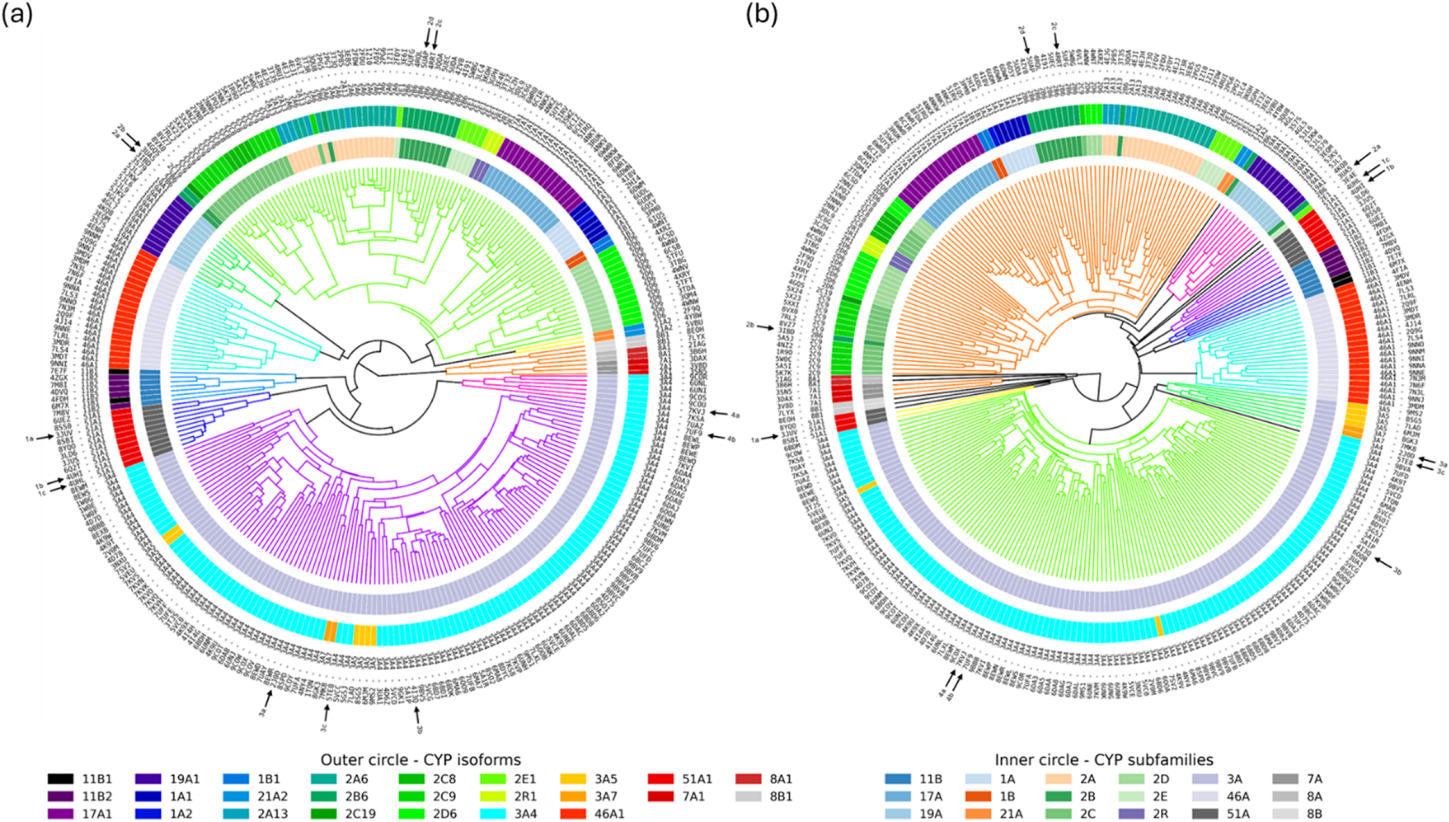
Radial dendrograms of human cytochrome P450s based on: (a) backbone similarity and (b) binding site vector similarity RMSD matrices. Each branch represents a single structure, with outer labels indicating the corresponding PDB code and CYP isoform. Outer ring colors correspond to isoforms, inner ring colors to CYP subfamilies. Dendrogram branch colors are automatically assigned to highlight clusters of higher similarity; branches outside the similarity cutoff are shown in black. Arrows labeled 1–4 indicate selected examples discussed in detail in the main text. High-resolution figures of both panels can be found in Supporting Information as Figures S5 and S6, respectively.

The dendrograms in Figure 5 reveal structural organization patterns among human CYPs, enabling a comparison of backbone and binding site similarities. Many CYP structures clustered consistently across both metrics, generally reflecting sequence-based relationships. For example, CYP3A4 structures are grouped together, sharing the cluster with CYP3A5 and CYP3A7. Similarly, CYP2 family members were located within one dendrogram branch.

Nonetheless, discrepancies among the dendrograms emerged. To elucidate the structural basis of these differences, four interesting examples are highlighted in Figure 5 (arrows and labels), with corresponding structural alignments provided in Figure 6.

**Figure 6:**
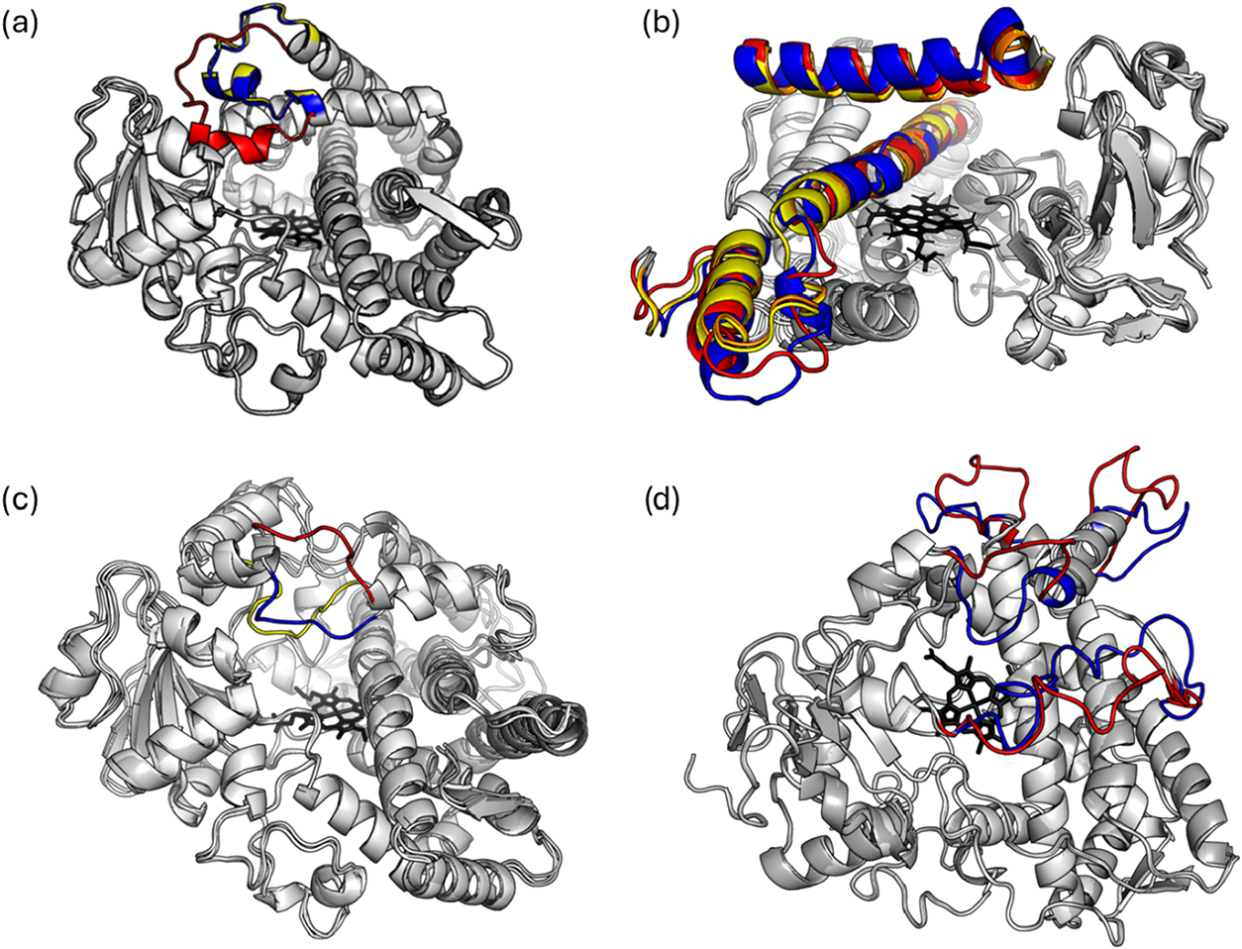
Superimposed structures illustrating cases where backbone and binding site-based similarity diverged: (a) CYP51A1 structures: 3JUV (1a, red), 4UHI (1b, blue), 4UHL (1c, yellow); (b) CYP2B6 structures: 3IBD (2a, red), 3UA5 (2b, blue), 4RRT (2c, yellow), 5UAP (2d, orange); (c) CYP3A4 structures: 2J0D (3a, red), 4I3Q (3b, blue), 5TE8 (3c, yellow); (d) CYP3A4 structures: 7KVJ (4a, red), 7UF9 (4b, blue). Overlapping structural regions are shown in different shades of gray, while divergent regions are highlighted in individual colors.

In Figure 6a, the CYP51A1 structures corresponding to branches 1a, 1b, and 1c in Figure 5 are shown. In the backbone-based dendrogram, these structures cluster together, with 1b and 1c more closely associated with each other than with 1a. In contrast, the binding site-based dendrogram strongly separates 1a from 1b and 1c. Structural inspection reveals that a helix–loop region adjacent to the binding pocket exhibits notable conformational variation: in 1b and 1c, this region is closely superimposed, whereas in 1a, it adopts a different arrangement. This variation has a relatively minor effect on overall backbone similarity but strongly influences binding site vector-based grouping.

In Figure 6b, the structures corresponding to branches 2a–2d in Figure 5 are shown, all belonging to CYP2B6. In the backbone-based dendrogram, these structures occupy the same major branch, with 2a/2b and 2c/2d positioned on closer subbranches. In contrast, the binding site-based dendrogram reveals a different arrangement: 2c and 2d remain closely associated, 2b retains placement within the same major branch but displays greater divergence, and 2a is assigned to a distinct branch. Structural overlays confirm these observations, with high overlap between 2c and 2d, whereas in 2a and 2b the helices are shifted and the loops adopt different structural arrangements, changing the binding site characteristics.

The third example (Figure 6c) highlights CYP3A4 structures, where missing residues were reconstructed using PDBFixer. These correspond to arrows 3a–3c in Figure 5. In the backbone-based dendrogram, the structures cluster within the main CYP3A4 branch. In the binding site-based dendrogram, however, 3b remained in the main CYP3A4 branch, while 3a grouped with CYP3A5 and CYP3A7, and 3c formed its own cluster. Structural overlays indicate that this divergence arises from a flexible loop above the heme, a region reconstructed by PDBFixer, which significantly impacts binding site geometry while having less effect on backbone-based grouping. This loop variation may result from reconstruction uncertainties and may not reflect the true conformation.

The final example depicts the reverse, less common situation: structures that appeared more similar to each other by binding site analysis than by backbone comparison. These correspond to CYP3A4 structures shown with arrows 4a and 4b in Figure 5. In the backbone-based dendrogram, the structures occupied two separate branches due to three flexible loops that adopt distinct conformations, as illustrated in Figure 6d. In contrast, binding site-based clustering indicated high similarity, as most of these loops were shielded from the binding pocket by the I helix, minimizing their impact on the local environment.

Overall, both dendrograms show similar organization, consistent with sequence-based relationships, while binding site vector analysis captures fine details within specific regions that are otherwise obscured in whole-structure comparisons. At the same time, this sensitivity highlights the importance of structural accuracy: as shown in Figure 6c, reconstruction of missing residues can alter binding site geometry and, consequently, vector-based clustering. Similar effects may occur in lower-resolution experimental structures, where the positional uncertainty can bias the resulting vectors.

### 3.2 Case Study 2: Ensemble-Averaged Cytochrome P450 Structural Similarity Derived from MD Simulations

Case Study 2 examined cytochrome P450s using conformational ensembles from molecular dynamics simulations. For eight human and 15 plant CYPs, pairwise similarities were computed based on sequence, backbone, and binding site overlap. In contrast to Case Study 1, structural similarities here were averaged across the entire conformational ensemble, as detailed in section 2.8.2. Figure 7 presents the resulting similarity trees.

**Figure 7:**
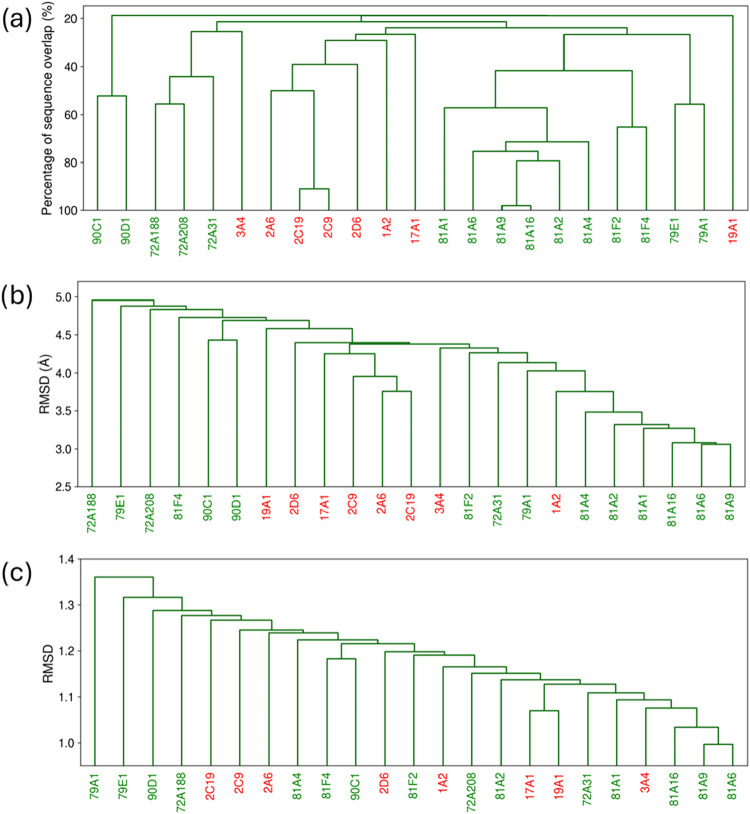
Dendrograms for eight human CYPs (labeled in red) and 15 plant CYPs (labeled in green) based on (a) sequence identity, (b) backbone similarity RMSD matrix, and (c) binding site vector similarity RMSD matrix.

#### 3.2.1 Human CYPs

Considering the human CYPs, the backbone-based dendrogram reflects sequence similarity with only minor variations in branching order. Within our selected set, four CYPs belong to the CYP2 family. Of these, three that cluster most closely based on sequence similarity also maintain the closest relationship in the backbone similarity analysis. In both trees, CYP17A1 exhibits equivalent grouping to the CYP2 family members, and CYP19A1, CYP3A4 and CYP1A2 remain the most divergent.

The vector-based dendrogram distributes human CYPs among the plant CYPs, capturing their diversity more effectively than overall fold or sequence similarities. A striking difference compared with the other dendrograms is observed in the close grouping of CYP17A1 and CYP19A1. Their grouping based on binding site properties corresponds to their high selectivity for steroid substrates involved in human endogenous metabolism,^39–42^ suggesting a similarly shaped active site.

Furthermore, within our set, the remaining six CYPs account for the majority of the human xenobiotic metabolism. To examine their functional overlap, we assessed their similarity based on the number of substrates they share, using the Human P450 Metabolism data from Rendić.^43^ For ease of comparison, in Figure S7, a substrate overlap-based similarity tree is provided alongside sequence-based, backbone-based, and binding site-based dendrograms for xenobiotic human CYPs only.

Consistent with the substrate-based grouping, the vector-based dendrogram captures the functional relationships. In both, CYP3A4 and CYP1A2 appear most closely associated, CYP2A6 is the most distant, and CYP2C9, CYP2C19, and CYP2D6 occupy intermediate, similarly spaced positions. In contrast, the backbone-based dendrogram reflects functional relationships less consistently yet more closely than the sequence-based grouping, which shows the weakest correspondence to function.

#### 3.2.2 Plant CYPs

Turning to the remaining 15 CYPs, distinct trends emerge, depending on the metric of comparison. In some cases, binding site similarity closely aligns with both sequence-based and backbone-based similarity, while in others, no clear correspondence is observed. Of the 15 enzymes analyzed, six belong to the CYP81A subfamily and two to the CYP81F subfamily. Deviations from strict family level grouping are already apparent in the backbone-based dendrogram and become more pronounced for CYP81As at the subfamily level when binding site similarity is considered. A similar pattern is seen for other CYP pairs belonging to the same family (CYP79, CYP90) or subfamily (CYP72A), where relationships progressively weaken from sequence similarity to binding site similarity. Only CYP81F2 and CYP81F4 remain closely associated across the metrics. While very little is known about the typical substrates of these CYPs, the binding site (dis)­similarity may offer indications about their substrate range and function.

#### 3.2.3 Binding Site Vector Dendrograms with Varying Feature Weights

Finally, to evaluate how specific binding site features influence enzyme clustering, similarity dendrograms were generated with different feature weightings. Figure 8 shows dendrograms based on vector length alone, equal weighting of length and charge (as in Figure 7b), and charge alone. Two additional dendrograms with a 50% greater emphasis on either shape or charge are presented in Figure S8.

**Figure 8:**
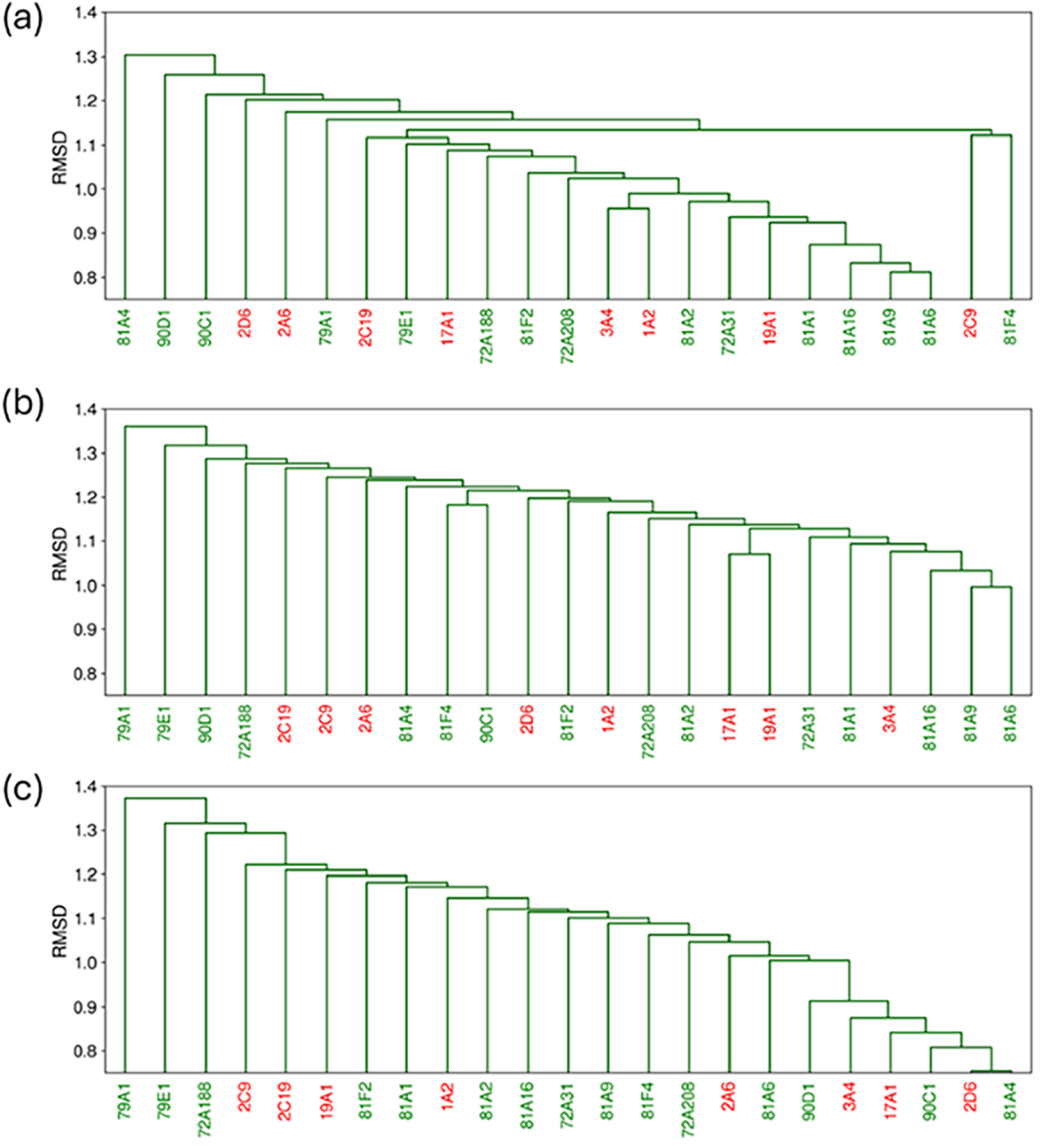
Dendrograms for eight human CYPs (labeled in red) and 15 plant CYPs (labeled in green) based on (a) binding site shape, (b) binding site shape and charge and (c) binding site charge.

CYPs that cluster closely in shape-based similarity dendrograms exhibit binding sites of similar vector lengths, whereas those clustering closely in charge-based dendrograms show similar binding site charge distributions. In contrast, the most diverse CYPs across the similarity trees show increasingly pronounced differences in these parameters. This trend is illustrated in Figure S9, which highlights the most extreme cases of shape and charge similarity (the right-most and left-most CYP pairs in Figures 8a,c), showing that closely related CYP pairs exhibit lower per-vector fluctuations in either length or charge compared with more distantly related pairs.

Comparing CYP groupings across the dendrograms reveals several trends. First, CYP81A6, CYP81A9, and CYP81A16 cluster together in panel b primarily due to binding site shape similarity, as seen in panel a, rather than charge similarity shown in panel c. Second, CYP17A1 and CYP19A1 do not exhibit strong similarity in panels a and c individually but show increased clustering affinity when shape and charge contributions are combined, indicating equal influence of both properties. Finally, CYP79A1 and CYP79E1 are distinguished mainly by charge specificity, with both the combined and the charge dendrogram placing them as the most distinct CYPs.

It is also informative to relate these clustering trends to known ligand binding behavior. For example, CYP3A4 and CYP1A2, which share a substantial fraction of known substrates, cluster more closely in the shape-based dendrogram than in the charge-based representation, although their electrostatic profiles are not markedly dissimilar.^44,45^ This suggests that the geometric compatibility of the binding site plays a dominant role in their overlapping substrate profiles. CYP1A2 is known to preferentially metabolize smaller, rigid, and often planar molecules, whereas CYP3A4 accommodates a much broader range of substrates through conformational plasticity. The observed shape similarity likely reflects those CYP3A4 conformations that can present a binding cavity comparable in size and geometry to those of CYP1A2, enabling productive binding of shared ligands.

To conclude case study 2, ensemble-averaged backbone similarity largely reflects sequence-based relationships with only minor overlaps with vector-based groupings. In contrast, binding site vectors reveal more distinct groupings that capture the functional diversity across CYPs.

### 3.3 Case Study 3: Binding Site Conformational Diversity and Overlap across Cytochrome P450s Derived from MD Simulations

Case Study 3 analyzed the same set of CYPs using the MD-generated conformational ensembles as in Case Study 2, but with a focus solely on binding site analysis. The large number of simulation frames was reduced to a smaller set of representative structures through two rounds of clustering, as described in Section 2.8.3. This approach gives a more detailed examination of pairwise CYP similarities that were averaged in previous case study. The proportion of conformations of each CYP populating the resulting clusters is shown in Figure 9.

**Figure 9:**
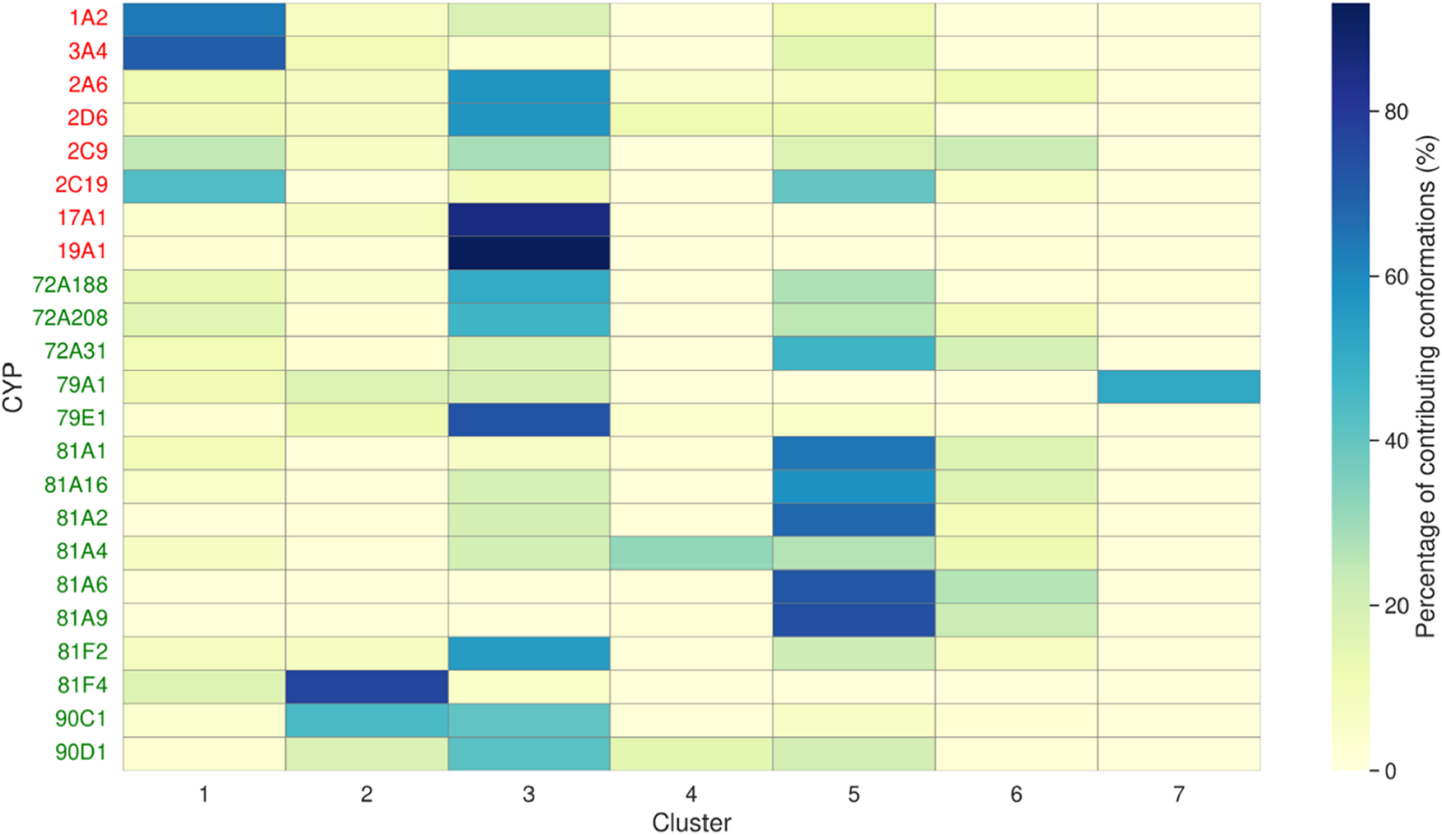
Heatmap showing the distribution of CYP conformations across clusters. Rows correspond to CYP isoforms (human CYPs are labeled in red, plant CYPs are labeled in green), and columns to the seven clusters. Color intensity reflects the percentage of conformations of a single CYP assigned to each cluster, with darker shades indicating higher contributions. Representative binding site structures for each cluster are shown in Figure S10.

Cluster populations reveal the conformational diversity of CYP binding sites with most isoforms contributing to multiple clusters. This is in line with the conformational heterogeneity typically associated with these enzymes.^46^ Nevertheless, certain clusters are taxon-specific: cluster 1 is populated mainly by human CYPs, while cluster 5 is populated mainly by plant CYPs. This separation suggests species-specific binding site conformations, as expected from the distinct metabolism pathways in humans and plants.^47–49^

To further interpret the functional similarity of specific clusters, we rely mainly on the knowledge of human CYPs, since only little is known about the substrates and functions of the plant ones.

The first example that illustrates the reported CYP functionality is found in cluster 3, where conformations of CYP17A1 and CYP19A1 are predominantly present. These isoforms are exclusively involved in steroid metabolism, and the fact that nearly all of their conformations fall within a single cluster suggests that cluster 3 represents a binding site conformation well suited for steroid-like molecules. This cluster is highly populated with other human and plant CYPs. Among them, CYP79E1 has the largest proportion of its conformations in this cluster, suggesting behavior similar to that of CYP17A1 and CYP19A1. Interestingly, CYP79E1 shares its only known function, tyrosine *N*-monooxygenase activity, with CYP79A1.^50^ They overlap in cluster 3, as well as in cluster 2, indicating shared functional characteristics, yet the majority of conformations observed for CYP79A1 belong to cluster 7. This distribution implies a functional overlap between the two enzymes, which includes tyrosine metabolism, while still reflecting potential differences in substrate preferences.

Second, in cluster 1, the vast majority of CYP3A4 conformations cluster together with many CYP2C9 and CYP2C19 ones, in agreement with their relative ability to metabolize large, bulky molecules. This cluster also includes CYP1A2, an enzyme typically associated with smaller, apolar ligands, making its coclustering with the more flexible and promiscuous CYPs unexpected.^51^ Such a grouping confirms the high average similarity previously observed between CYP1A2 and CYP3A4, suggesting that the substrate flexibility of CYP3A4 extends to a significant portion of CYP1A2’s ligand space (see Figure S7).

In conclusion, the conformational distribution in Case Study 3 explains the basis of the average similarities observed in Case Study 2, offering a richer, complementary perspective on CYP binding site relationships.

### 3.4 Cross-Comparison of Case Study Results

The structural comparisons made in the case studies of the previous sections are based either on static structures as in case 1 or on conformational ensembles as in cases 2 and 3. The statically observed CYPs include simulated ones. Here, we compare how these shared CYPs are grouped across the different analyses.

#### 3.4.1 Human CYPs Backbone-Based Similarity

Backbone similarity trees derived from static structures (Figure 5a) and MD ensembles (Figure 7b) show comparable results and correspond to sequence-based relationships. In both analyses, CYP2C9, CYP2C19, and CYP2A6 form a cluster, while CYP19A1 and CYP3A4 appear as distinct members. The main difference is the placement of CYP17A1 and CYP1A2, whose affinities to the CYP2 family are reversed between the two trees.

#### 3.4.2 Human CYPs Binding Site-Based Similarity

The relationships among CYPs based on binding site properties show partial overlap across approaches.

In the static structure comparison (Figure 5b), CYP2C9 and CYP2D6 are spread between other CYPs, consistent with their distribution across clusters from the dynamic study (Figure 9). This indicates that the diversity of their binding sites is reasonably captured by the available experimental structures, making them good representatives of the corresponding conformational ensembles. A similar observation can be made for CYP3A4, which displays distinct binding site characteristics in both approaches.

However, CYP2C19, which shares the largest number of conformations with CYP3A4 in the MD-based study, is represented by only a single crystal structure that does not cluster closely with any of the CYP3A4 structures. The same limitation applies to CYP1A2, making it difficult to draw conclusions about their conformational flexibility from Case Study 1.

Among all approaches, only the binding site vector similarity derived from MD simulation conformations grouped CYPs functionally, in line with known substrates. This finding further implies that the currently available experimental structures may not sufficiently capture the conformational diversity required to reveal such relationships. It raises a broader question: how many experimental structures are necessary to reliably represent the conformational space of a given CYP? For some CYPs, the available data appear adequate, while for others, they fall short.

#### 3.4.3 Plant CYPs

In Case Study 1, each plant CYP isoform was represented by a single modeled structure. Despite this limitation, several patterns were consistent with the dynamic observations. For example, members of the CYP81A family formed a common branch in backbone-based dendrograms both in static (Figure S3) and dynamic (Figure 7b) analyses. In vector-based dendrograms (Figures S4 and 7c), this family showed an internal variation: CYP81A6, CYP81A9, and CYP81A16 are grouped closely, CYP81A4 is consistently distinct, but the placement of the remaining isoforms diverged.

Additional correspondences across methods were observed for CYP79E1 and CYP72A188, which were isolated in both backbone- and binding site-based dendrograms, regardless of whether static or dynamic data were used.

In contrast, some plant CYPs displayed discrepancies between static and dynamic analyses. CYP81F2 and CYP81F4 appeared highly similar in static dendrograms but less so in dendrograms that include dynamic ensembles, whereas the divergence between CYP90C1 and CYP90D1 became more pronounced when dynamics were considered.

Based on insights from the human CYPs, we propose that dynamic data, especially binding site-focused comparisons, are likely to capture functional relationships of plants CYPs better.

### 3.5 Further Applications

#### 3.5.1 Systems Beyond Cytochrome P450s

In this work, we demonstrated the use of binding site vectors at the hands of the Cytochrome P450 superfamily of enzymes. In the current example, the heme iron offered itself as a natural pocket anchoring point, which needs to be carefully designed for other proteins. This could be, e.g., a central point in the active site, a specific catalytic residue, or another strongly conserved residue. With an appropriate anchor, the approach can be readily transferred to other proteins, particularly those whose binding sites are largely occluded from the bulk solvent. This feature highlights a key advantage of the binding site vectors: their ability to precisely define, characterize, and quantitatively compare pockets of interest. While existing methods efficiently identify and compare large numbers of pockets at once, they are often less suited either for fine-grained topological comparisons (e.g., fpocket) or for the analysis of cavities that are shielded from the solvent and thus difficult to access using a solvent accessible surface area approach (e.g., ProBiS^52^).

#### 3.5.2 Ligand Binding Modes

The same concept used to generate binding site vectors can be applied to capture the surface of a bound ligand. The ligand can be described by the same initial set of vectors as the binding site: the vectors originate from the same anchoring point and extend in the same directions but terminate at the ligand surface at the point farthest from the origin. For all vectors that intersect with the ligand, the resulting lengths need to be shorter than their corresponding binding site vectors. In cases in which the ligand lies in close proximity to a protein residue, the charges assigned to the ligand vectors are expected to be complementary to those of the corresponding binding site vectors. Representing the ligand in such a way enables direct evaluation of the compatibility of this exact binding mode, in this specific orientation, with any pocket of interest. Conceptually, this constitutes a binary docking criterion: rather than assessing docking pose similarity via RMSD, the combination of ligand and binding site vectors yields an efficient accept or reject outcome for a given ligand pose.

As a proof of concept, we created *ligand vectors* for the fluconazole binding mode found in the crystal structure with CYP3A4 (PDB ID: 6MA7). The resulting binding site vectors, ligand vectors, and associated surfaces are collected in Figure S11. The vector-defined binding environment of fluconazole is shown in Figure 10.

**Figure 10:**
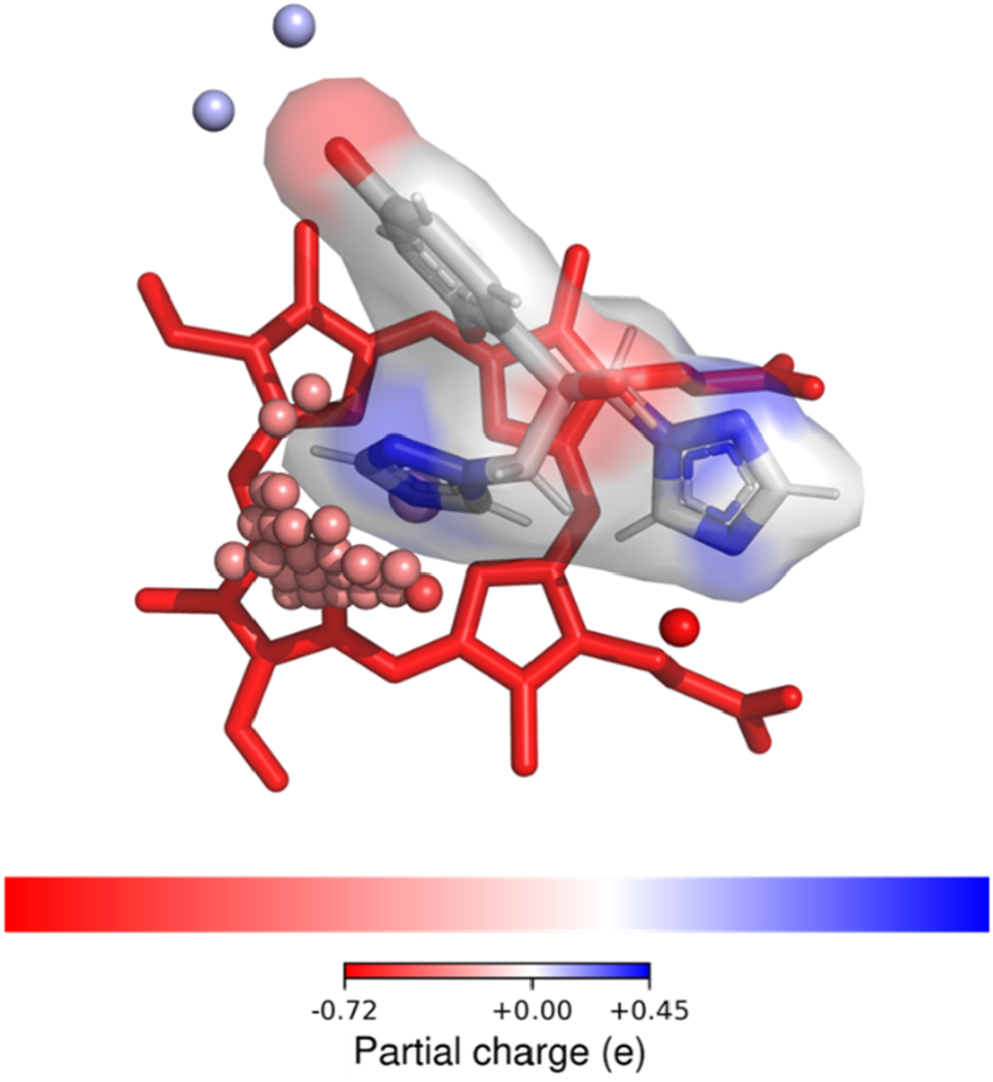
Fluconazole and its surrounding protein environment within the CYP3A4 binding pocket. Fluconazole is shown as sticks, with its surface and charge defined by the end points of the ligand vectors. Spheres indicate the first intersection points of the binding site vectors with CYP3A4, colored by the partial charge of the corresponding protein atoms. Only the spheres in close proximity to the ligand surface are shown, highlighting complementary charges between the two that support ligand binding.

The generated ligand vectors were used to evaluate ligand-mode fitting within MD-derived conformational ensembles of CYP3A4, CYP2C9, CYP2C19, and CYP1A2. Fluconazole provides a particularly suitable showcase for this analysis, as it is a known inhibitor of the first three isoforms.^53^ In the selected pose, the closest ligand atom lies 2.2 Å from the heme iron, consistent with typical inhibitor binding modes. For the collection of 15625 conformations of each CYP, the number of conformations in which the ligand vectors could be geometrically accommodated by the binding site vectors was determined, allowing up to 10% of the ligand vectors to deviate from perfect overlap by as much as 1 Å. Under these criteria, the ligand was compatible with 1,177 conformations of CYP3A4, 333 conformations of CYP2C9, and 669 conformations of CYP2C19, while no compatible conformations were identified for CYP1A2, in agreement with the known inhibition/binding profile of fluconazole.

#### 3.5.3 More Complex Binding Scenarios

In addition, binding site vectors can be used to investigate more complex binding scenarios. For example, water-mediated ligand binding^54^ can be explicitly incorporated by including selected water molecules as a part of the binding pocket, allowing the resulting vectors to capture solvent contributions to pocket geometry and electrostatics. A similar strategy can be applied to multiple-ligand binding, as observed for ketoconazole,^7^ caffeine,^51^ or aflatoxin B^55^ in CYP3A4. In such cases, one ligand can be included in the binding site representation, enabling the vectors to characterize the remaining pocket space and assess its suitability for accommodating an additional ligand.

### 3.6 Limitations

While the binding site vectors offer a simple characterization of the shape of an active site, there are several inherent limitations that should be considered when applied.

First, comparison of binding pockets critically depends on accurate structural alignment of the analyzed systems. This is particularly important for molecular dynamics (MD) trajectories where numerous conformations amplify alignment errors.

The second point arises from the vector-based representation itself: once a vector intersects with an atomic surface, no further sampling along that direction is performed. Consequently, regions of the pocket that are geometrically occluded—such as subpockets located behind bulky residues or secondary-structure elements—may not be captured. This issue can be mitigated by generating multiple vector sets anchored at different positions within the binding site, including within occluded regions.

Third, the electrostatic component of the descriptor relies on partial charges derived from static force fields. In reality, charge distributions may adjust to local environments or ligand binding events, which are not explicitly modeled here.

Finally, the method effectively captures systems governed by conformational selection, as it samples pre-existing conformations of the binding site. However, it does not account for induced-fit mechanisms, where the protein undergoes substantial structural rearrangements *upon* ligand binding.

## 4 Conclusions

In this study, we introduce *binding site vectors*, a computational framework for a high-resolution comparison of the structural and electrostatic properties of macromolecular binding sites. The method itself is conceptually simple yet powerful, providing a direct and quantitative means to assess binding site similarity. By encoding both geometric and electrostatic features, it can resolve subtle local differences that often drive functional specificity. As a result, we can capture the binding site flexibility of a single protein by analyzing multiple conformational states, as well as compare binding sites across different proteins in a consistent and interpretable manner.

We tested the methodology for cytochrome P450 enzymes. Binding sites of over 600 CYP structures were systematically characterized. A closer examination was performed on a subset of 23 CYPs using a broad ensemble of conformations that were generated by MD simulation.

Comparing CYP similarity based on binding site properties versus overall fold and sequence shows that our approach captures the structural-functional landscape of CYPs most effectively. Although static structure analysis provided valuable insights and enabled the examination of numerous isoforms, far more than is currently feasible with MD simulations, the use of full conformational ensembles revealed additional functional tendencies that remained hidden in static structures.

Taken together, we propose binding site vectors as the most detailed approach for comparing binding sites, yielding similarity groups that align with functional relationships in systems governed by the conformational selection model. We further recommend employing molecular dynamics simulations to generate multiple conformations, providing a representative data set that captures protein dynamics and enables functional relationships to be observed more clearly than with static experimental structures.

## Supplementary Material



## Data Availability

The data underlying this study are openly available in Zenodo at 10.5281/zenodo.17472839 (accessed on 29 October 2025), which include topologies, coordinates, and input files to perform all molecular simulations, as well as the scripts used to perform the analyses described in this work. The method is implemented as program pocket in the gromos++ suite of programs http://(www.gromos.net) as well as scripts gen_kuvek_bp_descr.py and cmp_kuvek_bp_descrs.py in the Chemical Data Processing Toolkit (CDPkit) (https://github.com/molinfo-vienna/CDPKit).

## Notes

Human CYPs with PDB IDs of analyzed structures; plant CYPs with UniProt codes of analyzed structures; phylogenetic tree of human cytochrome P450s; phylogenetic tree of plant cytochrome P450s; backbone-based similarity tree of plant cytochrome P450s; binding site vectors-based similarity tree of plant cytochrome P450s; backbone-based similarity tree of human CYPs; binding site vectors-based similarity tree of human cytochrome P450s; similarity trees of xenobiotic human cytochrome P450s; weighted dendrograms for eight human CYPs and 15 plant CYPs; binding site vectors fluctuations for shape and charge only examples; representative binding site structures for the seven obtained clusters; ligand and binding site vectors for CYP3A4-bound fluconazole (PDF)
