# Supplementary material for: Binding Site Vectors Enable Mapping of Cytochrome P450 Functional Landscapes
Source: J Chem Inf Model. 2026 Jan 26;66(3):1604–16. doi: 10.1021/acs.jcim.5c02705 (PMC12892331; doi:10.1021/acs.jcim.5c02705)

Binding Site Vectors Enable Mapping of Cytochrome P450 Functional Landscapes

Tea Kuvek,[1,2] Zuzana Jandová[3], Klaus-Juergen Schleifer[4], Chris Oostenbrink[1,2*]

[1]Institute for Molecular Modeling and Simulation, BOKU University, Muthgasse 18, 1190 Vienna, Austria

[2]Christian Doppler Laboratory for Molecular Informatics in the Biosciences, BOKU University, Muthgasse 18, 1190 Vienna, Austria

[3]Boehringer Ingelheim International GmbH, Dr.-Boehringer-Gasse 5-11, 1121 Vienna, Austria

[4]BASF SE, Carl-Bosch-Strasse 38, 67056 Ludwigshafen, Germany

*Email: chris.oostenbrink@boku.ac.at

Contents

Table S1. Human CYPs with PDB IDs of analysed structures.

Table S2. Plant CYPs with UniProt codes of analysed structures.

Figure S1. Phylogenetic tree of human cytochrome P450s.

Figure S2. Phylogenetic tree of plant cytochrome P450s.

Figure S3. Backbone-based similarity tree of plant cytochrome P450s.

Figure S4. Binding site vectors-based similarity tree of plant cytochrome P450s.

Figure S5. Backbone-based similarity tree of human CYPs.

Figure S6. Binding site vectors-based similarity tree of human cytochrome P450s.

Figure S7. Similarity trees of xenobiotic human cytochrome P450s

Figure S8. Weighted dendrograms for eight human CYPs and fifteen plant CYPs

Figure S9. Binding site vectors fluctuations for shape and charge only examples

Figure S10. Representative binding site structures for the seven clusters

Figure S11. Ligand and binding site vectors for CYP3A4-bound fluconazole

Supplementary tables

Table S1. Human CYPs with PDB IDs of analysed structures. Structures having IDs marked with * were simulated.

CYP	PDB ID
**1A1**	6UDM, 6DWM, 6O5Y, 6UDL, 6DWN, 4I8V
**1A2**	2HI4*
**1B1**	6IQ5, 3PM0
**2A6**	3T3Q, 1Z10, 4RUI, 2PG5, 3EBS, 4EJJ, 2FDU, 1Z11, 2FDV*, 2PG6, 2FDW, 2FDY, 2PG7, 3T3R
**2A13**	4EJI, 4EJG, 3T3S, 4EJH, 2P85
**2B6**	3QOA, 4ZV8, 4I91, 5UDA, 4RQL, 5WBG, 5UEC, 4RRT, 3QU8, 5UAP, 5UFG, 3UA5, 3IBD
**2C8**	2NNI, 1PQ2, 2NNH, 2VN0, 2NNJ
**2C9**	7RL2, 8VX0, 5X23, 5A5I, 5X24, 5XXI, 5A5J, 8VZ7, 5W0C*, 1R9O, 6VLT, 5K7K, 4NZ2
**2C19**	4GQS*
**2D6**	4XRZ, 4WNT, 3QM4, 3TDA, 4WNU, 6CSD, 5TFT, 3TBG, 4WNV, 6CSB, 5TFU, 4XRY, 4WNW, 2F9Q*
**2E1**	3E6I, 3E4E, 3KOH, 3LC4, 3GPH, 3T3Z
**2R1**	3DL9, 3C6G, 3CZH
**3A4**	7KVO, 6DAG, 9BV9, 5VCC, 4D75, 4I4H, 4K9T, 7KVH, 1W0G, 5VCD, 9BV7, 6BDK, 7KS8, 4I3Q, 3UA1, 7KVS, 4NY4, 9COS, 9COT, 8EWS, 7UAZ, 6UNG, 6UNI, 6DA8, 7UFD, 7UFC, 2V0M, 9BVA, 4D7D, 5TE8, 9COU, 8EWR, 6OOA, 8SO1, 9COR, 6BCZ, 9MS1, 5G5J, 9BV6, 8SPD, 6BDM, 4I4G, 4K9U, 7KVI, 3NXU, 1W0F, 8EWN, 5VCE, 6DAA, 7KVN, 9BV8, 6BD5, 7UFB, 5A1R, 7UFE, 8DYC, 8EXB, 6OO9, 1TQN, 6UNH, 7KSA, 6DA3, 5VC0*, 6BD6, 7UFF, 6BD8, 7UFA, 9BVC, 6UNK, 9BBB, 6UNL, 6MA8, 6MA6, 9GK1, 6UNE, 7KVQ, 8SO2, 6OOB, 9COV, 8EWQ, 9COX, 2J0D, 4K9X, 6DAL, 9BV5, 8EWD, 6BDI, 7KVM, 7UF9, 4K9V, 7KVJ, 6DAB, 8EWM, 1W0E, 7UAY, 6MA7, 3TJS, 6UNM, 6UNJ, 9BVB, 5A1P, 6DA5, 6BD7, 6DA2, 4K9W, 7KVK, 5VCG, 6DAC, 8EWL, 6DAJ, 8EWE, 6BDH, 4D78, 9COY, 7LXL, 9COW, 8EWP, 7KVP, 4D6Z
**3A5**	5VEU, 8SG5, 7SV2, 9MS2, 7LAD, 6MJM
**3A7**	7MK8, 8GK3
**7A1**	3DAX, 3SN5, 3V8D
**8A1**	2IAG, 3B6H
**8B1**	8EOH, 7LYX
**11B1**	7E7F, 6M7X
**11B2**	7M8I, 4DVQ, 4FDH, 4ZGX, 7M8V
**17A1**	6WW0, 3SWZ, 3RUK*, 6WR1, 4NKZ, 6WR0, 5UYS, 6CIZ, 8FDA, 4NKW, 6CHI, 4NKY, 5IRV, 6CIR, 5IRQ, 4NKX, 4NKV
**19A1**	3EQM, 4KQ8, 3S7S, 3S79*, 4GL7, 5JL9, 5JL7, 5JKV, 4GL5, 5JL6, 5JKW
**21A2**	4Y8W, 5VBU
**46A1**	3MDT, 4FIA, 9NNI, 7LRL, 2Q9F, 7N3L, 9NNA, 7LS4, 7LS3, 9NNO, 3MDR, 7N3M, 2Q9G, 9NNE, 3MDM, 3MDV, 4J14, 4ENH, 7N6F, 9NNJ, 9NNM
**51A1**	8SBI, 8YQO, 3LD6, 4UHL, 3JUV, 8SS0, 3JUS, 4UHI, 6UEZ, 6Q2T

Table S2. Plant CYPs with UniProt codes of analysed structures. CYPs marked with * were simulated.

CYP	UniProt code	CYP	UniProt code	CYP	UniProt
**51G1**	Q9SAA9	**72A188***	M1CIZ9	**88A108**	A0A5B8ND22
**71A1**	P24465	**72A208***	M1AUM0	**88D6**	B5BSX1
**71A2**	P37118	**72A219**	H2DH21	**89A2**	Q42602
**71A4**	P37117	**72A397**	A0A0S2IHL2	**89A9**	Q9SRQ1
**71A6**	O04164	**72A552**	A0A481NR20	**90A1**	I1IUJ6
**71A8**	Q42716	**72C1**	Q9SHG5	**90A3**	B8BJ22
**71A9**	O81970	**73A1**	Q04468	**90A4**	Q5CCK1
**71A12**	O49340	**73A2**	Q9AR74	**90B1**	I1H7R8
**71A14**	P58045	**73A3**	P37114	**90B2**	B8AJL3
**71A15**	P58046	**73A4**	P48522	**90C1***	Q9M066
**71A16**	Q9FH66	**73A5**	P92994	**90D1***	Q94IA6
**71A18**	Q9SAB6	**73A9**	Q43067	**90D2**	A2WLP4
**71A19**	Q9T0K0	**73A10**	Q43033	**92C5**	A0A1D6HSP4
**71A20**	Q9T0K2	**73A11**	Q42797	**92C6**	A0A1D6F9Y9
**71A21**	Q9STL2	**73A12**	Q43240	**93B1**	P93149
**71A22**	Q9STL1	**73A13**	O24312	**93B2**	Q9XGT9
**71A23**	Q9STL0	**73A14**	Q96423	**93B16**	E9KBR8
**71A24**	Q9STK9	**73A16**	Q43054	**93C2**	Q9SXS3
**71A25**	Q9STK8	**73A19**	O81928	**93E1**	Q9XHC6
**71A26**	Q9STK7	**73A33**	Q94IP1	**93G1**	Q0JFI2
**71A27**	O65438	**73A100**	H2DH22	**93G2**	Q5VRI5
**71A28**	P58047	**75B1**	Q9SD85	**94A1**	O81117
**71AJ3**	C0SJS2	**75B2**	Q9SBQ9	**94B3**	Q9SMP5
**71AJ4**	C0SJS3	**75B3**	Q7G602	**98A1**	O48956
**71AN24**	A0A068Q609	**75B4**	Q8LM92	**98A2**	O48922
**71AP13**	A0A068Q721	**75B137**	A0A4D6Q415	**98A3**	O22203
**71AU50**	A0A068Q5V6	**75B138**	A0A4D6Q414	**98A8**	Q9CA61
**71AV1**	Q1PS23	**76AD1**	I3PFJ5	**98A9**	Q9CA60
**71AV8**	E1B2Z9	**76AH1**	S4UX02	**99A2**	Q7X7X4
**71AY1**	W8JDE2	**76AH3**	A0A0Y0GRS3	**99A3**	Q0JF01
**71AZ1**	D2CGS0	**76AH22**	A0A0C5Q4Y6	**701A6**	Q5Z5R4
**71AZ3**	A0A2Z5D850	**76AH24**	A0A0S1TP26	**701A8**	Q0DBF4
**71AZ4**	A0A2Z5D854	**76AH30**	A0A1Z3GBS4	**701A9**	Q5Z5S6
**71AZ6**	A0A2Z5D852	**76AJ1**	W0FKI0	**701A19**	Q5Z5R7
**71B2**	O65788	**76AK1**	A0A125QZE2	**703A2**	Q7EZR4
**71B3**	O65785	**76AK6**	A0A1D8QMD1	**704C1**	Q50EK3
**71B4**	O65786	**76AK7**	A0A1D8QMD2	**705A1**	Q0WQ07
**71B5**	O65784	**76AK8**	A0A1D8QMG4	**705A5**	Q9FI39
**71B6**	O65787	**76B6**	Q8VWZ7	**705A12**	Q9FH67
**71B7**	Q96514	**76B9**	Q14T82	**705A20**	Q9LJY7
**71B8**	P58048	**76B10**	D1MI46	**705A22**	Q9LJY5
**71B9**	O64718	**76B74**	A0A3Q9R4N5	**707A1**	Q949P1
**71B10**	Q9LVD2	**76C1**	O64636	**707A2**	K4CI52
**71B11**	P58049	**76C2**	O64637	**707A3**	Q9FH76
**71B12**	Q9ZU07	**76C3**	O64638	**707A4**	Q9LJK2

**71B13**	P58050	**76C4**	O64635	**707A5**	Q05JG2
**71B14**	P58051	**76M5**	Q6YTF5	**707A6**	Q09J78
**71B16**	Q9LTM7	**76M6**	Q6Z5I7	**707A7**	A2Z212
**71B17**	Q9LTM6	**76M7**	Q69X58	**708A2**	Q8L7D5
**71B19**	Q9LTM4	**76M8**	Q6YTF1	**709B1**	Q9ASR3
**71B20**	Q9LTM3	**77A4**	Q9LZ31	**709B2**	F4IK45
**71B21**	Q9LTM2	**78A5**	Q9LMX7	**709B3**	Q9T093
**71B22**	Q9LTM1	**78A6**	Q9ZNR0	**710A1**	O64697
**71B23**	Q9LTM0	**78A7**	Q9FIB0	**710A2**	O64698
**71B24**	Q9LTL8	**78A9**	Q9SLP1	**710A3**	Q9ZV29
**71B25**	Q9LTL2	**78A11**	Q7Y1V5	**710A4**	Q9ZV28
**71B26**	Q9LTL0	**79A1***	Q43135	**710A11**	Q1T7C2
**71B28**	Q9SAE3	**79A2**	Q9FLC8	**711A1**	B9DFU2
**71B29**	Q9SAE4	**79B1**	O81345	**714A1**	Q93Z79
**71B31**	Q9SCN2	**79B2**	O81346	**714A2**	Q6NKZ8
**71B34**	Q9LIP6	**79D2**	Q9M7B7	**714B1**	Q7XHW5
**71B35**	Q9LIP5	**79D4**	Q6J540	**714B2**	Q0DS59
**71B36**	Q9LIP4	**79E1***	Q43135	**714B3**	B6SSW8
**71B37**	Q9LIP3	**79F1**	Q949U1	**714C1**	B9GBJ9
**71B38**	Q9LXM3	**79F2**	Q9FUY7	**714C2**	Q2QYH7
**71BA1**	E3W9C4	**80B1**	O64899	**714C3**	B9G934
**71BE52**	A0A0S1TQ04	**80B2**	Q9FXW4	**714D1**	Q5KQH7
**71BE54**	A0A0N9HT29	**80G2**	A8CDR5	**716A1**	Q9LVY7
**71BL1**	F8S1H3	**81A1***	B6SYA2	**716A2**	A0A140JWM8
**71BL2**	X2JE85	**81A2***	A0A804M4E3	**716A12**	Q2MJ20
**71BL3**	G3GBK0	**81A4***	B6U863	**716A15**	F6H9N6
**71BL6**	A0A2H4DGV8	**81A6***	Q2LA61	**716A17**	A5BFI4
**71BQ4**	A0A067F4I6	**81A9***	B6ST45	**716A44**	A0A3Q7HBJ5
**71BQ5**	A0A5B8NEF2	**81A16***	B6SSF2	**716A46**	K4CEE8
**71C1**	Q43250	**81D1**	Q9FG65	**716A47**	H2DH16
**71C2**	Q43255	**81D11**	Q9LHA1	**716A52**	I7C6E8
**71C3**	P93703	**81E7**	Q6WNR0	**716A53**	I7CT85
**71C4**	Q43257	**81E8**	Q6WNQ8	**716A67**	Q2MJ21
**71CB1**	X2EW55	**81E9**	Q6WNQ9	**716A75**	A0A0B4L1W8
**71CD1**	A0A067GFT7	**81F1**	O65790	**716A81**	A0A0U2U8U5
**71CD2**	A0A5B8NBK9	**81F2***	Q9LVD6	**716A94**	A0A0S2II38
**71CU1**	A0A0N9HTU1	**81F3**	Q0WTF4	**716A141**	A0A1I9Q5Z0
**71D1**	I1TEM1	**81F4***	Q9SZU1	**716AC1**	A0A067ELB0
**71D12**	P98183	**81Q1**	Q33DY0	**716AD2**	A0A067DE75
**71D13**	Q9XHE7	**81Q2**	Q33DX9	**716B1**	Q50EK1
**71D15**	Q9XHE6	**82C2**	O49394	**716B2**	Q50EK0
**71D18**	Q6WKZ1	**82C3**	O49396	**716E26**	A0A3Q7HS74
**71D20**	Q94FM7	**82C4**	Q9SZ46	**719A1**	Q948Y1
**71D55**	A6YIH8	**82D33**	M1KXD0	**719A2**	Q50LH3
**71D94**	Q6WKZ0	**82D61**	A0A0N7F297	**719A3**	Q50LH4
**71D95**	Q6WKY9	**82D62**	M1KVN4	**719A5**	B5UAQ8
**71D178**	P0DO35	**82E3**	Q38Q84	**719A13**	B1NF19
**71D179**	P0DO36	**82E4**	A1YJE3	**719A14**	B1NF20
**71D180**	P0DO40	**82G1**	Q9LSF8	**719A21**	I3QBP4

**71D181**	P0DO41	**82N3**	L7X0L7	**719A23**	L7T8H2
**71D182**	A0A159AKG3	**82N4**	L7X3S1	**719A24**	L7T720
**71D313**	H2DH20	**82X1**	I3V6B7	**719A37**	A0A7T9QPT0
**71D351**	U5HKE8	**82X2**	I3PLR0	**719B1**	B1NF18
**71D445**	A0A165U5Z9	**82Y1**	I3PLR1	**720B1**	Q50EK6
**71DD6**	F8S1H8	**83A1**	P48421	**720B2**	Q50EK5
**71E1**	O48958	**83B1**	O65782	**724B1**	Q6F4F5
**71E7**	Q6XQ14	**84A1**	Q42600	**726A27**	A0A161GJD5
**71P1**	Q2QUC5	**84A4**	F4JW83	**734A1**	O48786
**71Z6**	A3A871	**85A1**	Q8GSQ1	**734A2**	Q6Z6D6
**71Z7**	Q6YV88	**85A2**	Q940V4	**734A4**	Q69XM6
**71Z16**	A0A1D6GQ67	**85A3**	Q50LE0	**734A5**	Q8LIF2
**71Z18**	B4FVP3	**86A2**	O23066	**734A6**	B9X287
**72A11**	Q9LUC9	**86A4**	Q9LMM1	**735A1**	Q9FF18
**72A13**	Q9LUC8	**86A7**	Q9CAD6	**735A2**	Q9ZW95
**72A14**	Q9LUC6	**86A22**	B3RFJ6	**736A12**	H2DH18
**72A15**	Q9LUC5	**87A3**	Q7XU38	**736A117**	A0A068Q6L2
**72A31***	A2WS96	**87D18**	K7NBR2	**749A22**	H2DH17
**72A63**	H1A981	**88A1**	Q43246	**750A1**	Q50EK4
**72A68**	Q2MJ19	**88A37**	A0A067DT54		
**72A154**	H1A988	**88A51**	A0A067E1K2		

Supplementary figures

Figure S1. Phylogenetic tree of selected human cytochrome P450s. Each branch represents a single sequence, with outer labels indicating the corresponding CYP isoform. Outer ring colors correspond to isoforms, inner ring colors to CYP subfamilies. Dendrogram branch colors are automatically assigned to highlight clusters of higher similarity; branches outside the similarity cutoff are shown in black.

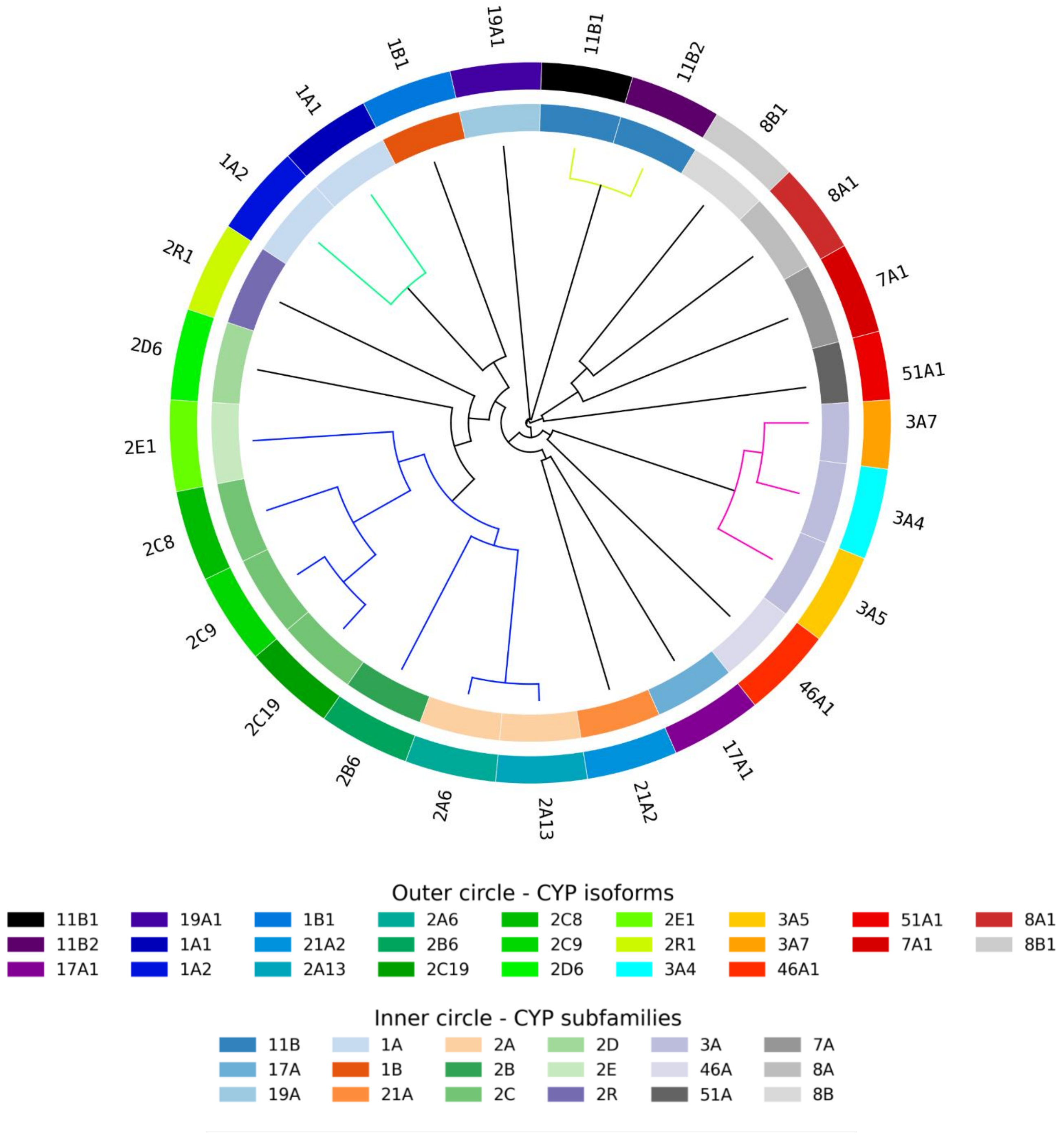

Figure S2. Phylogenetic tree of plant cytochrome P450s. Each branch represents a single sequence, with outer labels indicating the corresponding CYP isoform. Ring colors correspond to CYP families. Dendrogram branch colors are automatically assigned to highlight clusters of higher similarity; branches outside the similarity cutoff are shown in black.

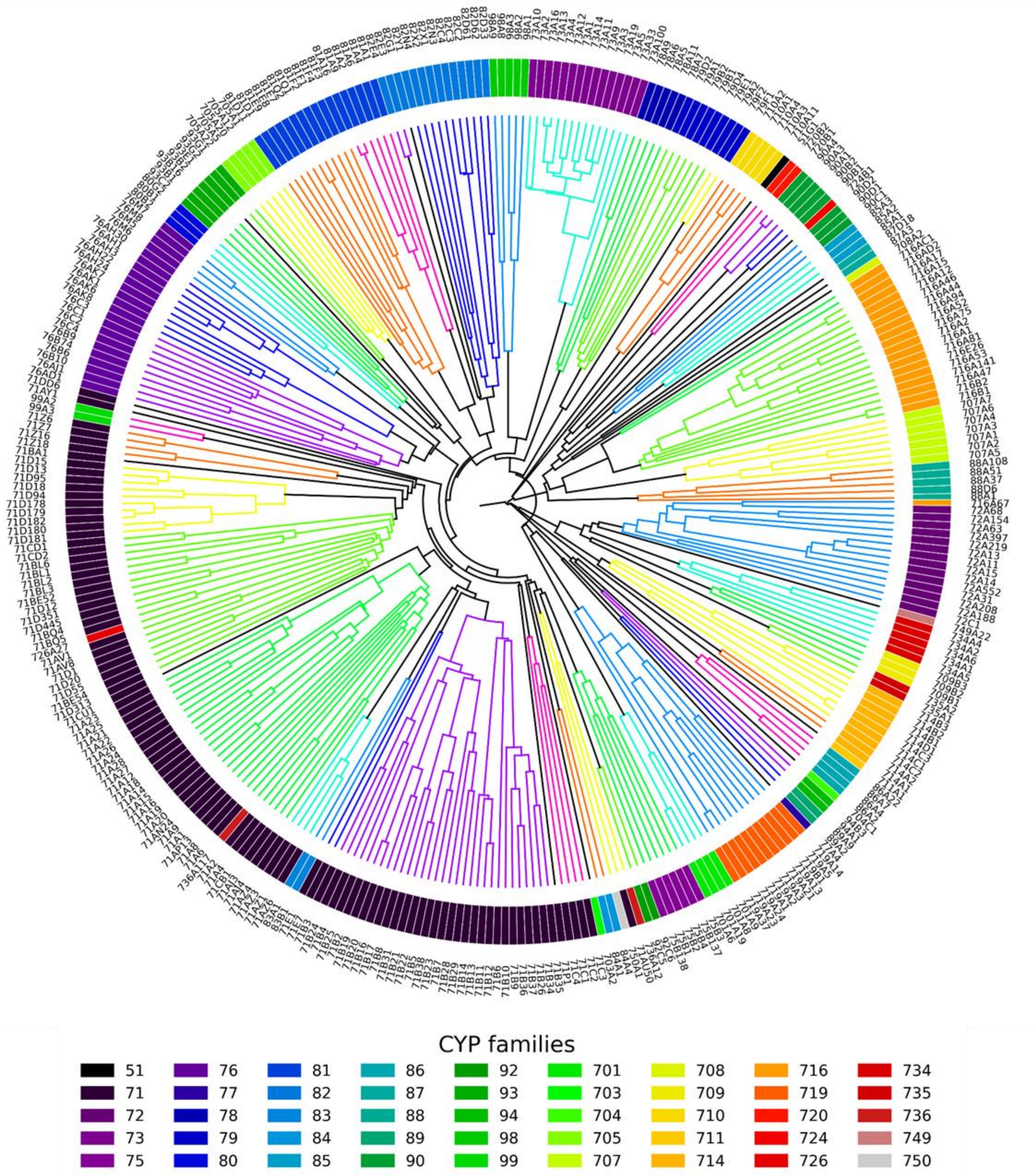

Figure S3. Backbone-based similarity tree of plant cytochrome P450s. Each branch represents a single structure, with outer labels indicating the corresponding UniProt code and CYP isoform. Ring colors correspond to CYP families. Dendrogram branch colors are automatically assigned to highlight clusters of higher similarity; branches outside the similarity cutoff are shown in black.

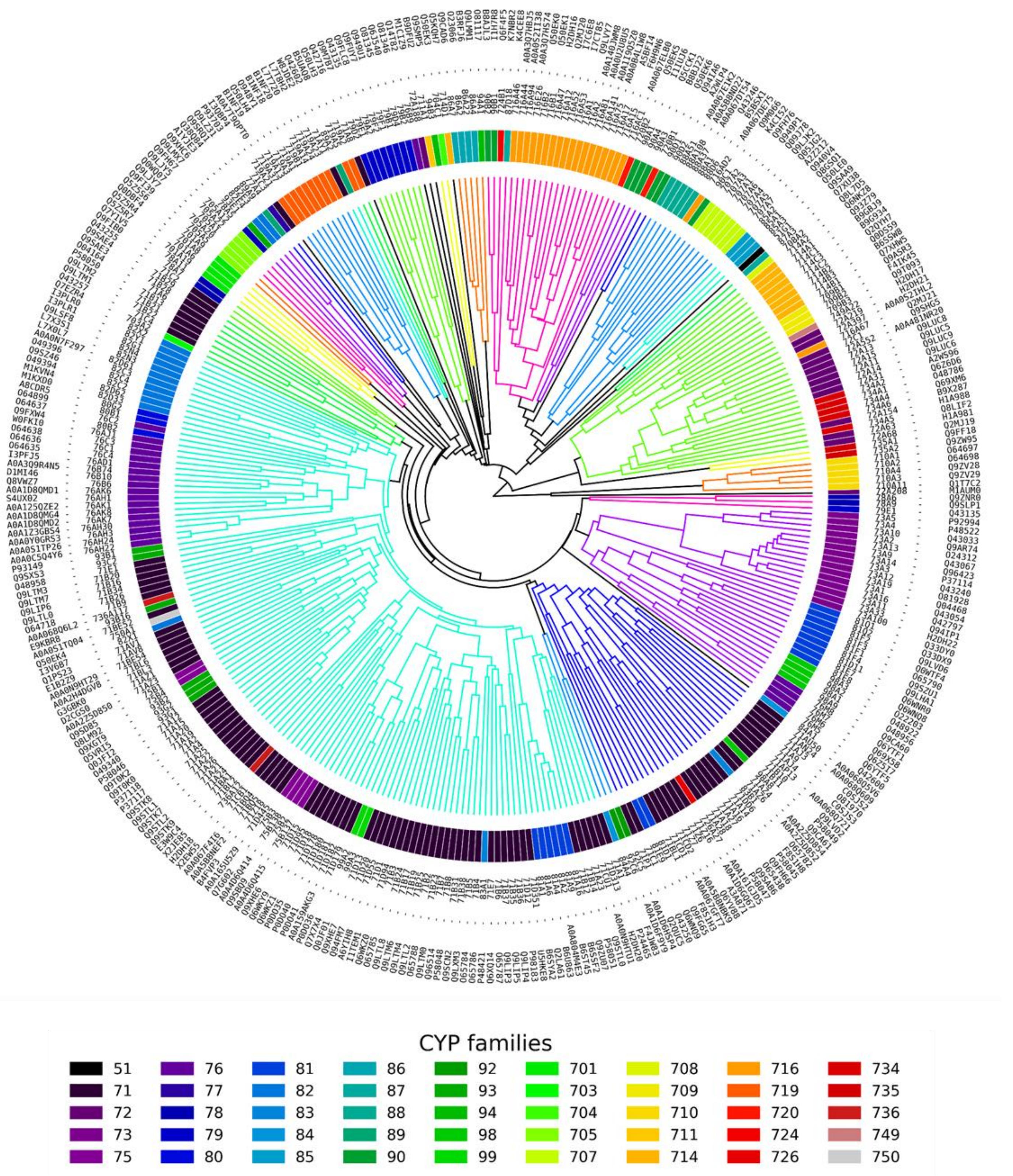

Figure S4. Binding site vectors-based similarity tree of plant cytochrome P450s. Each branch represents a single structure, with outer labels indicating the corresponding uniprot code and CYP isoform. Ring colors correspond to CYP families. Dendrogram branch colors are automatically assigned to highlight clusters of higher similarity; branches outside the similarity cutoff are shown in black.

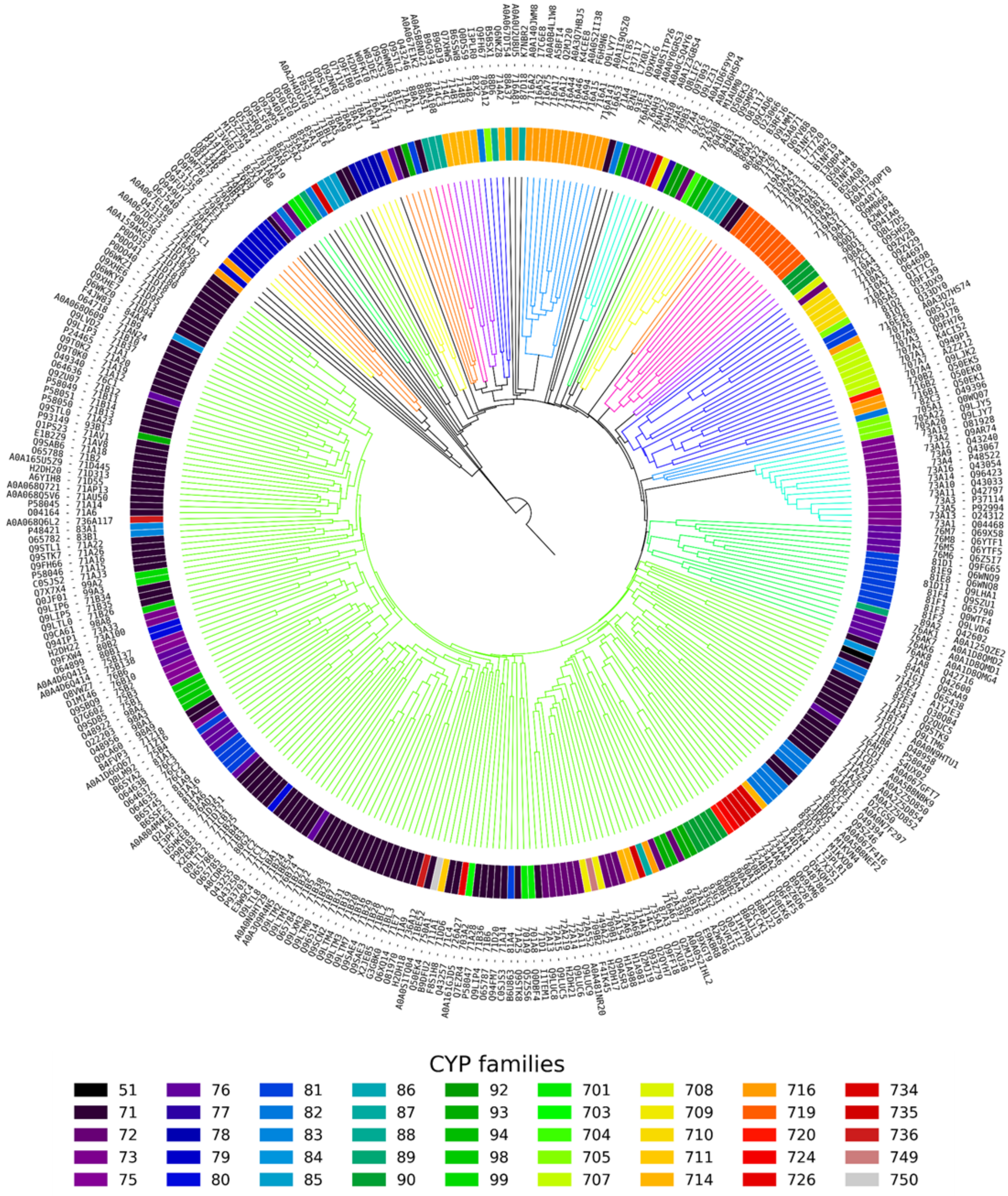

Figure S5. Backbone-based similarity tree of human CYPs. High-resolution version of figure 4a of the main manuscript.

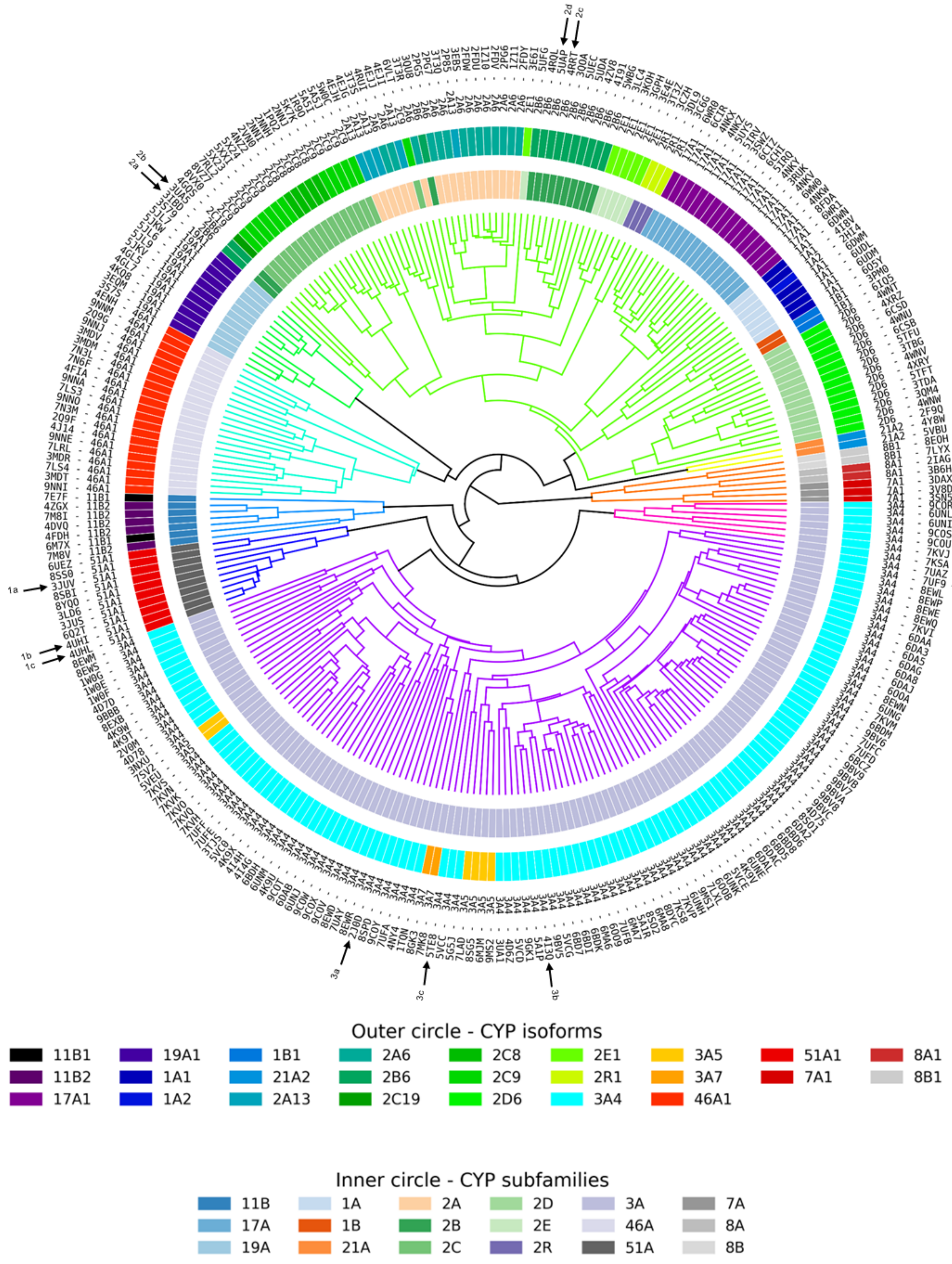

Figure S6. Binding site vectors-based similarity tree of human cytochrome P450s. High resolution version of figure 4b of the main manuscript.

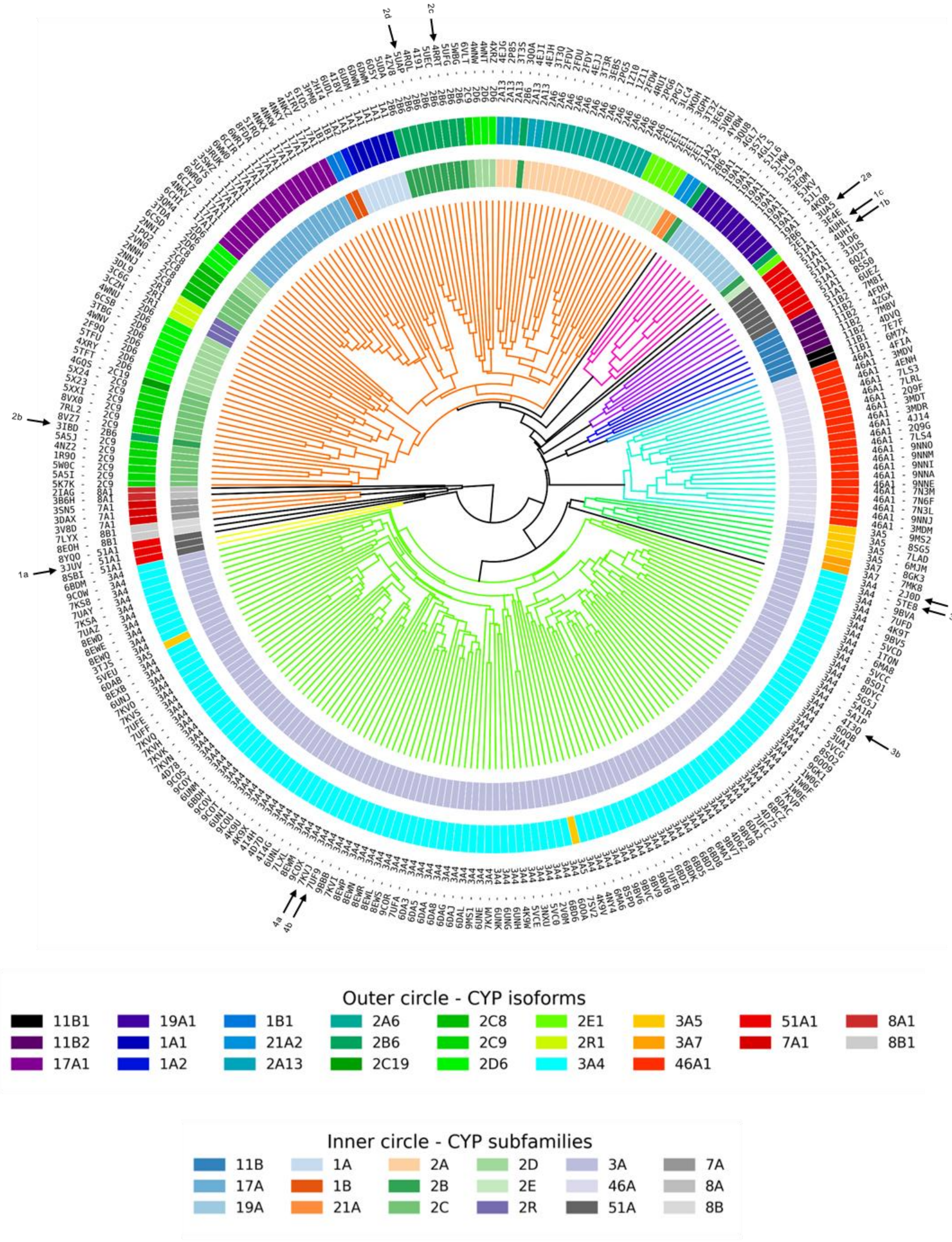

Figure S7. Similarity trees of xenobiotic human cytochrome P450s based on: a) sequence identity, b) backbone similarity RMSD matrix, c) binding site vectors similarity RMSD matrix and d) substrate overlap matrix. The overlap in panel d stands for shared number of substrates between two CYPs. Displayed substrate similarity was calculated as $1 - (\mathrm{overlap}/\mathrm{max\_overlap})$, where max_overlap is the highest number of shared substrates observed for any CYP pair. Numbers shown below each CYP's name indicate the number of substrates reported for that enzyme according to Rendić (Chem Res Toxicol 2015, 28, 38).

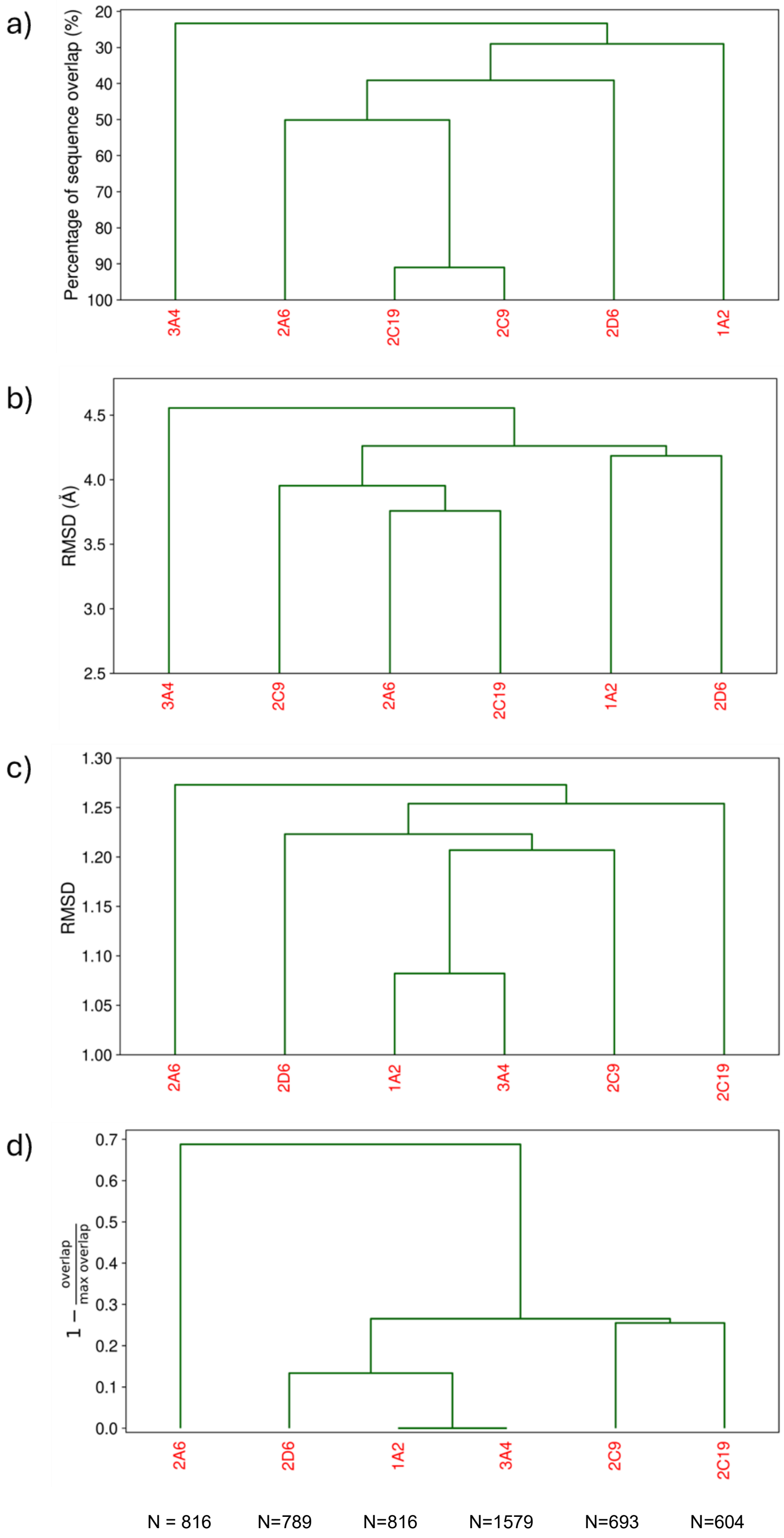

Figure S8. Dendrograms for eight human CYPs (labeled in red) and fifteen plant CYPs (labeled in green) based on binding site vectors with 50% greater emphasis on: a) 50% binding site shape and b) binding site charge.

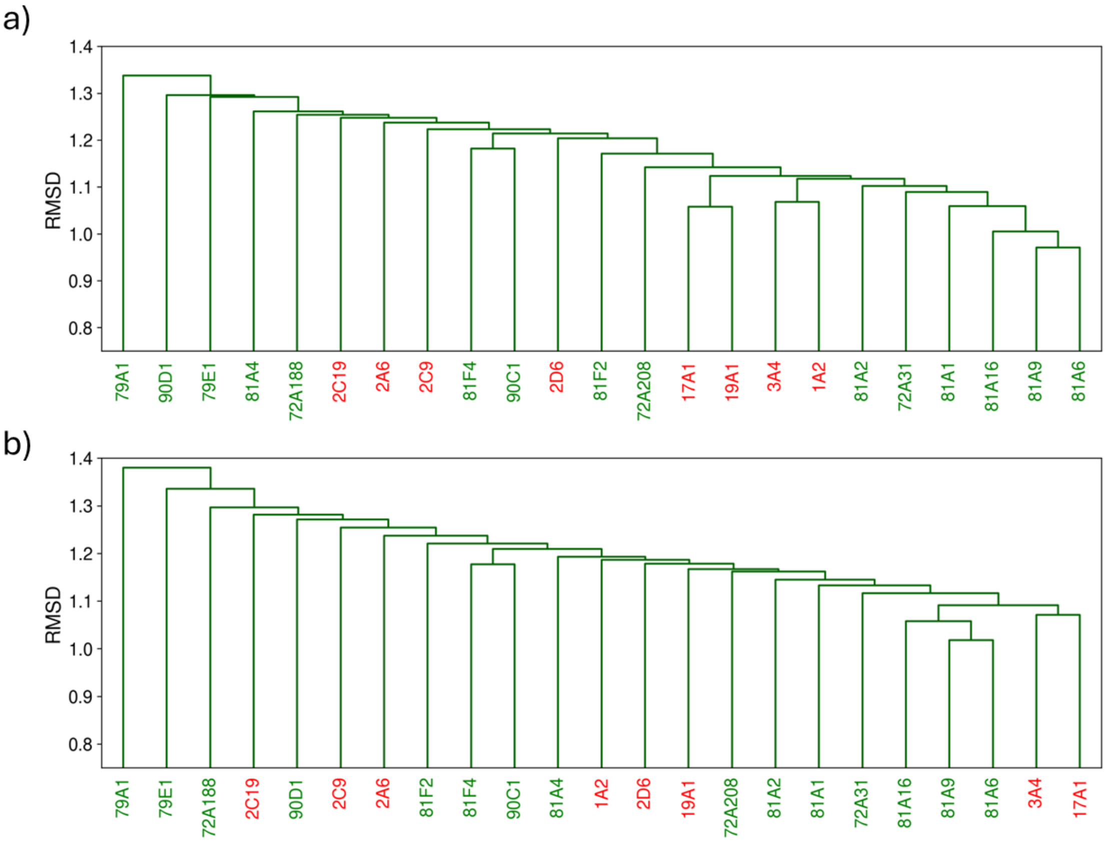

Figure S9. Binding-site hemispheres colored by root-mean-square fluctuation (RMSF) values for CYP pairs exhibiting the most similar and the most divergent binding-site shapes and charge distributions. The displayed RMSF values represent the average fluctuation of either the vector length or vector charge at each vector position relative to its mean value, calculated over the representative snapshots of the respective CYP pair. Panels 1a and 1b show RMSF values for vector lengths using a red color gradient, while panels 2a and 2b show RMSF values for vector charges using a green color gradient.

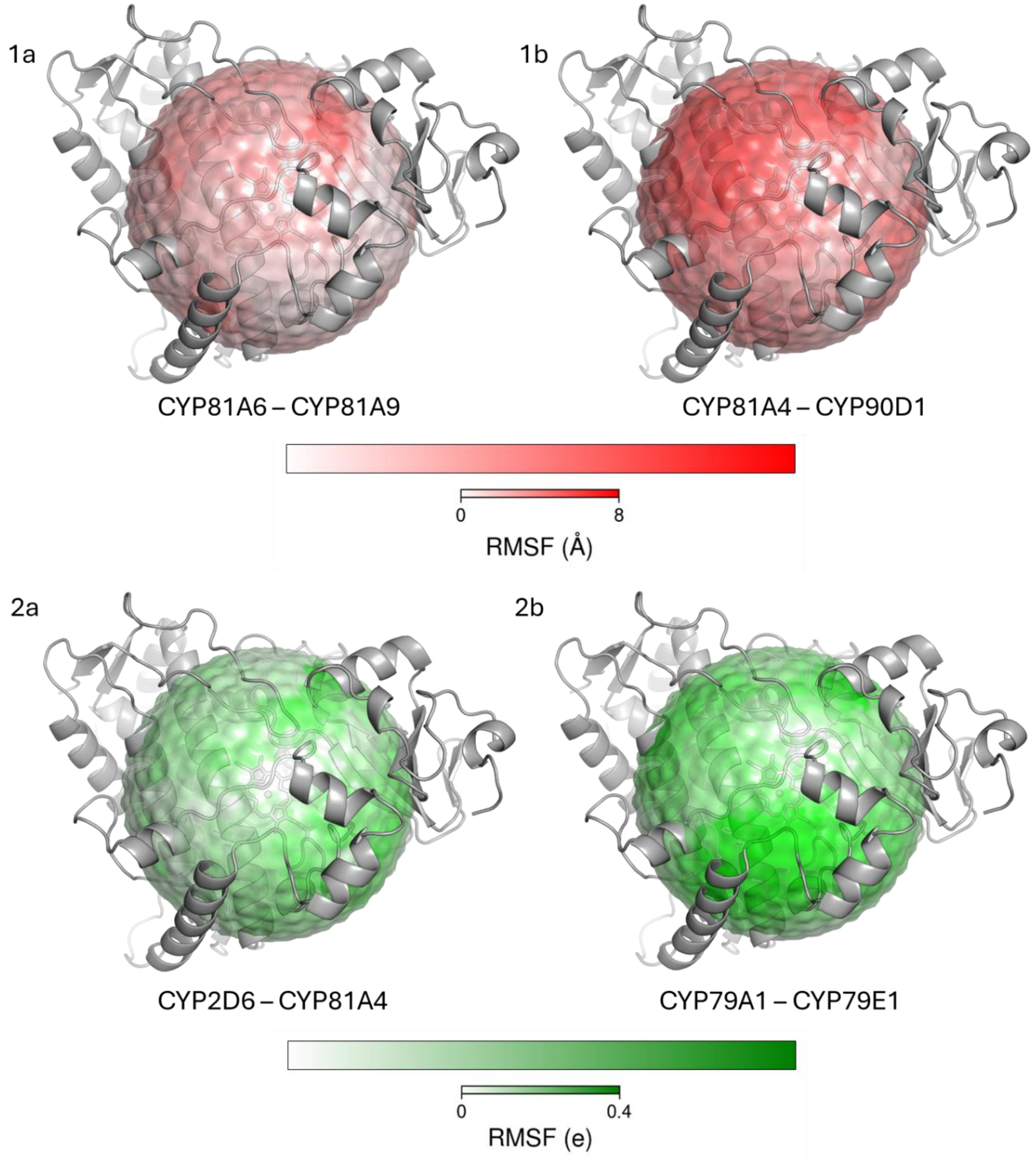

Figure S10. Representative binding site structures for the seven clusters identified in Case Study 3. Cluster numbers, shown in the upper-left corner of each panel, correspond to the clusters in Figure 8. The binding site surface is colored according to the partial charge on each vector with a gradient from red (negative) to white (neutral) to blue (positive).

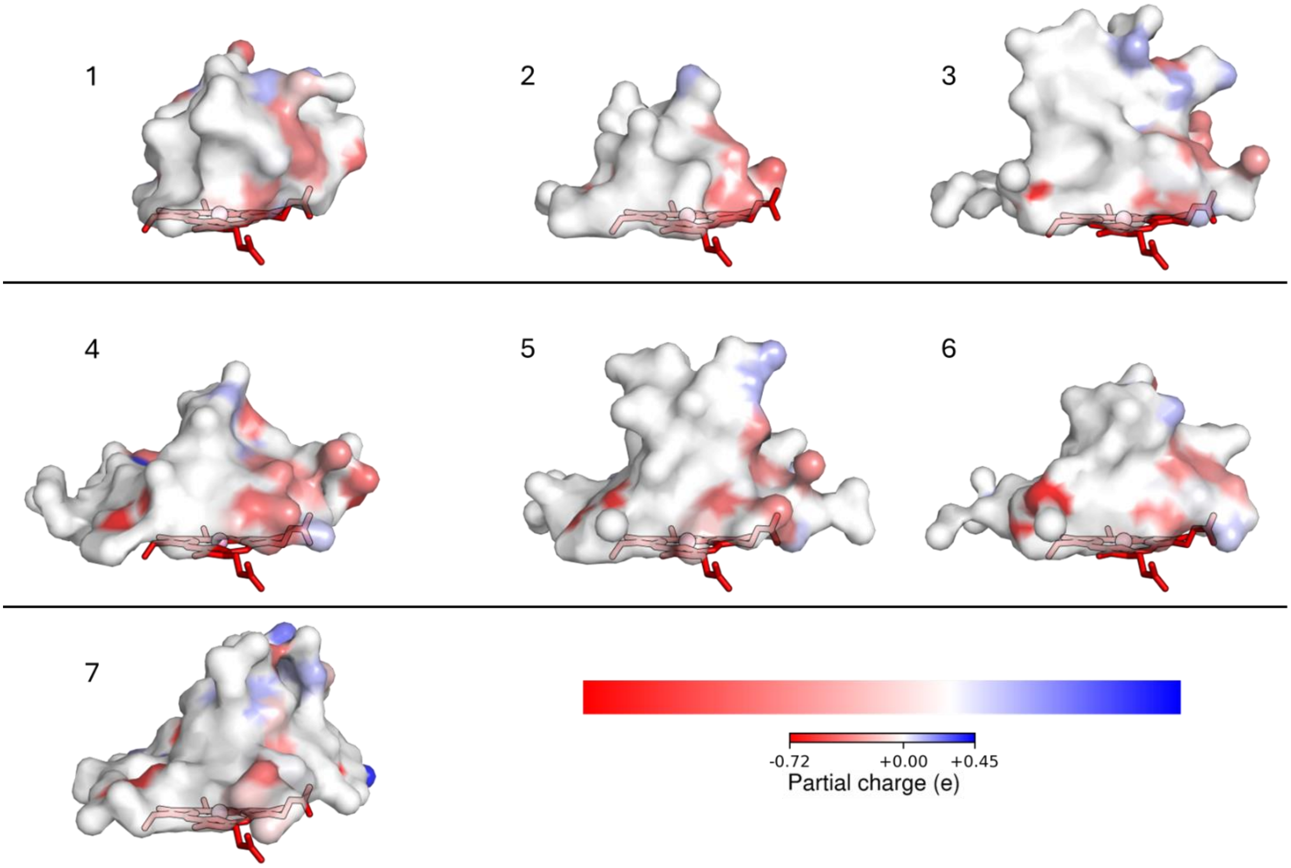

Figure S11. Crystal structure of CYP3A4 with bound fluconazole: (1) Ligand vectors, (2) Surface formed from ligand vectors, (3) Binding site vectors and (4) Surface formed from the binding site vectors. The presented surfaces are colored according to the partial charge on each vector with a gradient from red (negative) to white (neutral) to blue (positive).

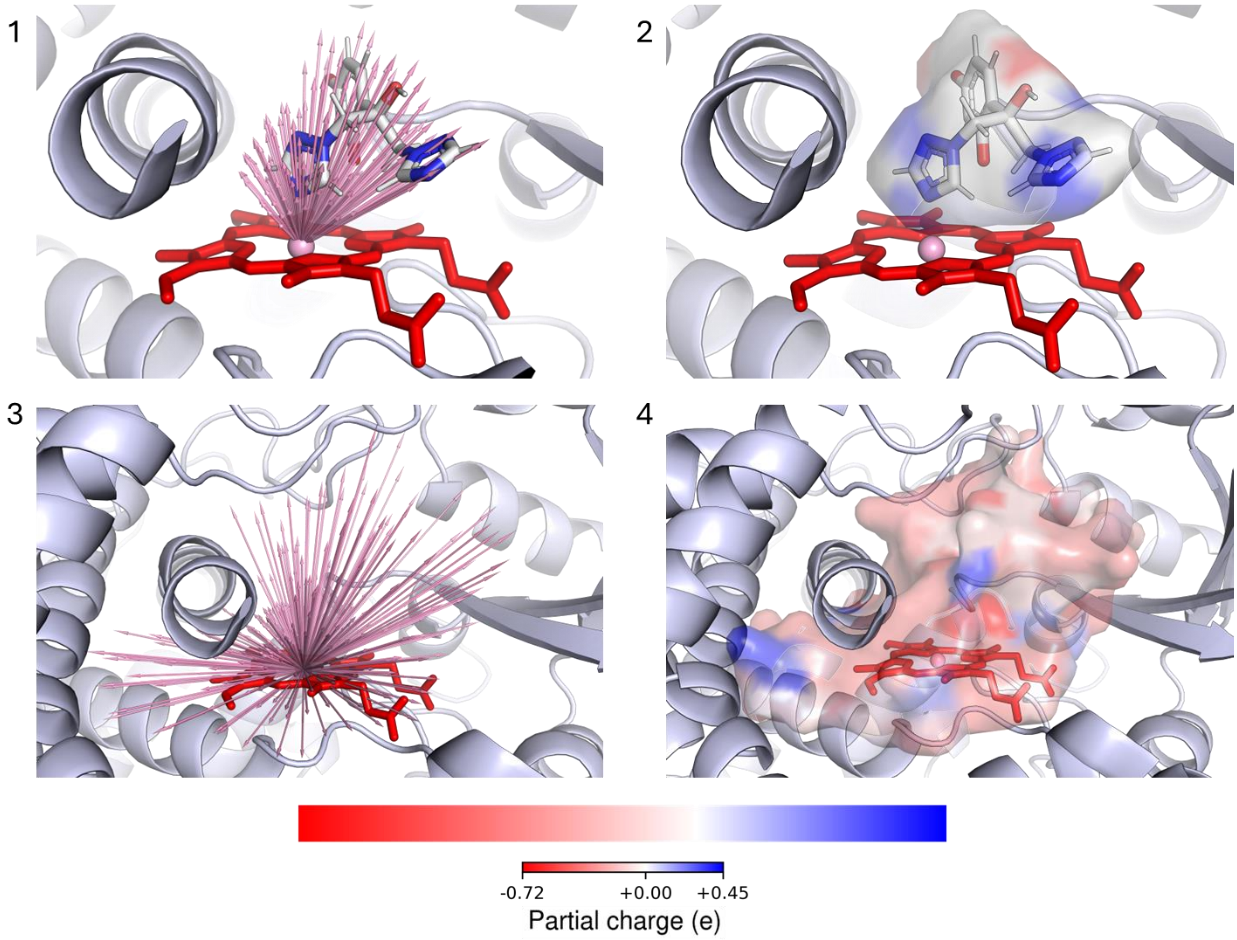

Supplement: Supplementary file 1 [file ci5c02705_si_001.pdf]
